# Cytokine production by bovine adipose tissue stromal vascular fraction cells upon *Neospora caninum* stimulation

**DOI:** 10.1038/s41598-024-58885-z

**Published:** 2024-04-10

**Authors:** Bárbara M. Oliveira, Beatriz Sidónio, Alexandra Correia, Ana Pinto, Maria M. Azevedo, Paula Sampaio, Paula G. Ferreira, Manuel Vilanova, Luzia Teixeira

**Affiliations:** 1 UMIB-Unidade Multidisciplinar de Investigação Biomédica, ICBAS-Instituto de Ciências Biomédicas de Abel Salazar, Universidade do Porto, Rua de Jorge Viterbo Ferreira, 4050-313 Porto, Portugal; 2 ITR-Laboratory for Integrative and Translational Research in Population Health, 4050-290 Porto, Portugal; 3 i3S-Instituto de Investigação e Inovação em Saúde, Universidade do Porto, Rua Alfredo Allen, 4200-135 Porto, Portugal; 4 ICBAS-Instituto de Ciências Biomédicas Abel Salazar, Universidade do Porto, Rua de Jorge Viterbo Ferreira, 4050-313 Porto, Portugal

**Keywords:** Immunology, Cytokines, Infection

## Abstract

In bovines few studies addressed the contribution of adipose tissue to the host immune response to infection. Here we evaluated the in vitro response of bovine adipose tissue stromal vascular fraction (SVF) cells to the protozoan parasite *Neospora caninum*, using live and freeze-killed tachyzoites. Live *N. caninum* induced the production of IL-6, IL-1β and IL-10 by SVF cells isolated from subcutaneous adipose tissue (SAT), while in mesenteric adipose tissue (MAT) SVF cell cultures only IL-1β and IL-10 production was increased, showing slight distinct responses between adipose tissue depots. Whereas a clear IL-8 increase was detected in peripheral blood leucocytes (PBL) culture supernatants in response to live *N. caninum*, no such increase was observed in SAT or MAT SVF cell cultures. Nevertheless, in response to LPS, increased IL-8 levels were detected in all cell cultures. IL-10 levels were always increased in response to stimulation (live, freeze-killed *N. caninum* and LPS). Overall, our results show that bovine adipose tissue SVF cells produce cytokines in response to *N. caninum* and can therefore be putative contributors to the host immune response against this parasite.

## Introduction

It has been increasingly recognized that besides its role in energy balance, the adipose tissue can contribute to the immune response to infection, as described for infections ranging from parasites to bacteria and viruses^1–3^. Many immune cell populations have been described in adipose tissue, whose phenotypes might be distinct from the same immune cells found in other tissues^4–6^. Macrophages are one of best studied immune cells due to their accumulation in adipose tissue in diet-induced obesity and their potential to secrete pro-inflammatory cytokines such as TNFα, IL-6, and IL-1β^4^. For example, recent in vitro studies have shown that SARS-CoV-2 virus can infect adipose tissue macrophages and induce the production of pro-inflammatory cytokines^7^. Lymphocyte cell populations are also found in adipose tissue, such as pathogen specific memory T cells, as shown for example in *Toxoplasma gondii* and *Yersinia pseudotuberculosis* murine models of infection^6^. On the other hand, immune cells have been shown to have other functions such as regulating thermogenesis^8,9^. This has accentuated the interest of studying the effect of infection in adipose tissue physiology^2,3,10^. Moreover, the adipose tissue is also a source of non-immune cells, such as mesenchymal stromal cells that also produce cytokines^11–13^. In cattle, we and others have previously described diverse lymphoid^14–16^ and myeloid populations^15–18^ present in bovine adipose tissue. However, to the best of our knowledge, no studies have yet explored the response of bovine adipose tissue stromal vascular fraction cells to infection. Nevertheless, exposure of adipose tissue explants to LPS from *Escherichia coli* resulted in increased expression of pro-inflammatory cytokines^19,20^.

*Neospora caninum* is an obligate intracellular protozoan parasite of the phylum Apicomplexa, closely related to *T. gondii*^21^. *N. caninum*, unlike *T. gondii*, is not considered a zoonotic protozoan^22^. Although *N. caninum* can infect many species, it assumes particular importance in cattle being the most frequent infectious agent detected upon bovine abortion in the 2000–2022 period worldwide^23,24^. Neosporosis leads to substantial economic losses in dairy and beef industry that have been estimated at over one billion dollars per year^21,25,26^. No commercial vaccine exists to prevent *N. caninum* infection^24^ and therefore a comprehensive understanding of the immune response to this parasite is highly desirable. In the murine model, we showed that infection with *N. caninum* significantly affected the frequency of leukocyte cell populations in adipose tissue^27,28^. Namely, increased number of macrophages, T-bet^+^ cells and regulatory T cells (Treg) were observed in the adipose tissue of the infected mice. Some alterations persisted even after the infection was cleared, including a higher Th1/Treg cell ratio^27^. At early time points upon the parasitic challenge, we also observed increased frequencies of cells producing IFN-γ, namely NK, NK T, TCRγδ^+^, and CD4^+^ and CD8^+^ TCRβ^+^ cells^28^. This response was largely abrogated in lethally susceptible IL-12/IL-23 p40-deficient mice^28^.

Interestingly, we observed that bovines seropositive to *N. caninum* (naturally exposed to the parasite) presented a higher frequency of macrophages and a lower frequency of a CD45-negative population with the CH138A^+^CD11b^−^MHC-II^−^ phenotype, in the subcutaneous adipose tissue (SAT), comparatively to seronegative ones^17^, hinting at the influence of infection in the bovine adipose tissue cell composition, as observed for the murine model. In cattle, the contribution of adipose tissue to the response to *N. caninum* infection and the interaction of this parasite with immune and non-immune cells in adipose tissue are unknown. Therefore, here, we aimed at characterising the potential contribution of stromal vascular fraction (SVF) cells present in bovine adipose tissue to an early host immune response to *N. caninum*. Total SVF cells, isolated from subcutaneous and mesenteric adipose tissue (MAT) of Holstein Friesian cows, were cultured with *N. caninum* tachyzoites (live and freeze-killed) and the levels of pro-and anti-inflammatory cytokines were assessed. Our results showed that bovine adipose SVF cells respond to both live and freeze-killed parasites with production of cytokines such as interleukin (IL)-6, IL-8, IL-1β and IL-10 with slight differences observed between subcutaneous and mesenteric adipose tissue. This may reflect the differences in the SVF composition of bovine adipose tissue from different anatomical locations that we previously reported.

## Methods

### Sample collection

Samples included in this study were randomly collected at a local slaughterhouse from 13 female Holstein–Friesian cattle (*Bos taurus*), slaughtered for human consumption, on six different occasions. Therefore, no animals were slaughtered or manipulated for research purposes and only by-products were recovered after slaughter of the animal. The authorization to use animal by-products was given by the competent national authority Direção-Geral da Alimentação e Veterinária (N.12.006.UDER: authorization number as user of by-products). The age of each individual animal is detailed in Supplementary Table S1. Sample size was determined with G* Power software^29^ using data from preliminary experiments. Samples were obtained using previously described procedures^14,17^. Briefly, samples of peripheral blood were collected, immediately after slaughter, at the time of bleeding procedure, from the jugular vein directly into tubes with EDTA (ethylene diamine tetraacetate, BD Vacutainer®, New Jersey, USA). Small fragments of SAT (removed from the flank region) and MAT (collected from the fat surrounding the mesenteric lymph nodes, avoiding the lymph nodes) were harvested immediately post-slaughter from the carcass and placed in Dulbecco’s Modified Eagle Medium (DMEM) supplemented with 100 units/mL penicillin, 100 μg/mL streptomycin, 250 ng/mL amphotericin B and 10 mM HEPES buffer (all from Sigma-Aldrich, St Louis, Missouri, USA). The tubes containing the adipose tissue samples were placed in a container with a warm water bath (38–39 °C) and transported straightaway to the laboratory for analysis. Blood was transported at room temperature.

### Evaluation of anti-*N. caninum* antibodies in blood serum

Screening of anti-*N. caninum* antibodies in bovine serum was done as previously described in detail^17^ using a commercial ELISA kit (ID Screen® *Neospora caninum* Indirect Multi-species, ID.Vet, Grabels, France). Only animals seronegative to *N. caninum* were included in this study.

### Isolation of bovine peripheral blood leukocytes

The isolation of peripheral blood leukocytes (PBL) was done as previously described^14,17^ with methodology similar to one previously described^30^. Whole blood was incubated in the same proportion with red blood lysis buffer solution [162.64 mM NH_4_Cl (Sigma-Aldrich), 9.98 mM Tris base (Merck, Darmstadt, Germany), pH = 7.2] for 10 min, with agitation, at room temperature. Cells were then passed through a 100-μm cell strainer, washed, and resuspended in complete RPMI medium: RPMI 1640 Medium supplemented with 10% foetal bovine serum (FBS) (FBS South America Premium, Ref. S181BH-500 from Biowest, Nuaillé, France), 85 units/mL penicillin, 85 μg/mL streptomycin, 62.5 ng/mL amphotericin B, 0.05 mM 2-mercaptoethanol and 10 mM HEPES (all from Sigma-Aldrich) after centrifugation at 300×*g* for 5 min.

### Isolation of stromal vascular fraction cells from bovine adipose tissue

The isolation of SVF cells was done by a previously described methodology, with slight modifications^14,17^. Briefly, 1–2 g of SAT or MAT were incubated with Hanks’ balanced salt solution supplemented with 10 mM HEPES (both from Sigma-Aldrich), 4% BSA (Biowest), and 0.125 mg/mL Liberase™ TL Research Grade (Roche Diagnostics, Risch-Rotkreuz, Switzerland) in a water bath at 37 °C during 60–90 min with manual agitation every 10 min. Digested samples were then passed through a 100-μm cell strainer and centrifuged at 280×*g* for 10 min at 4 °C. The pellet, corresponding to the SVF cells, was resuspended in complete RPMI medium.

### Parasites

*Neospora caninum* tachyzoites (NcT) from strain Nc-1 (ATCC™ 50,843) were isolated from infected VERO cell cultures as previously mentioned, with slight modifications^28,31,32^. Briefly, VERO cells infected with NcT were cultured at 37 °C and 5% CO_2_, in minimum essential medium with Earle’s salts (Corning, NY, Missouri, USA) supplemented with 2 mM L-glutamine, 200 units/mL penicillin and 200 μg/mL streptomycin (all from Sigma-Aldrich) and 10% FBS (Biowest), till 70% destruction of host cell monolayer. All the contents of the flask (the adherent cells collected using a cell scraper and the culture supernatants) were centrifuged at 1500×*g* for 20 min. The pellet was then passed through a 25G needle and washed three times in complete RPMI medium (all centrifugations were done at 1500×*g* for 20 min). The final pellet was resuspended in 3 mL of complete RPMI medium and passed through a PD-10 column filled with Sephadex G-25 M (GE Healthcare Life- Sciences, Freiburg, Germany). Freeze-killed NcT were prepared from suspensions of live tachyzoites (prepared as described above) resuspended in complete RPMI and kept at least one week frozen at − 80 °C, since others have previously shown that a 2 h freezing incubation at − 70 °C was enough to inactivate NcT^33^.

### Cell culture and stimulation with parasite

Stromal vascular fraction cells isolated from MAT and SAT as well as isolated bovine peripheral blood leukocytes were plated at 3 × 10^5^ cells/well in 96-well culture plates. Stimulation was done using live or freeze-killed NcT in cell/NcT ratios of 10:1, 5:1 or 1:1 for 4 and 12 h at 37 °C and 5% CO_2_. The ratios and time points were chosen to minimize host cell death since preliminary experiments showed that a ratio of cell/NcT ratio of 1:5 incubated for 12 h induced a significant reduction in host cell viability, as determined by flow cytometry analysis (Supplementary Fig. S1). As positive controls we used cells stimulated with 5 μg/mL of Lipopolysaccharides from *E. coli* O111:B4 (LPS, Sigma-Aldrich Cat. No. L4391), the same LPS used in Mukesh et al.^19^. Non-stimulated cells (incubated with complete RPMI alone) were used as negative controls. Samples from the same animals were analysed at 4 and 12 h, except for MAT where the number of cells recovered from 3 animals were not sufficient, and only the 12 h time point was done. After incubation, the plates were centrifuged at 366×*g*, for 15 min at 6 °C, and the supernatants (150 μL) were collected to evaluate by ELISA the production of cytokines. The pellets were either immediately used for cell cytospin or resuspended in QIAzol Lysis Reagent (QIAGEN sciences, MD, USA) and stored at − 80 °C until RNA extraction. Complete RPMI medium without cells was also incubated in the plates, and the supernatants were recovered to assess a putative source of cytokines independent of cells originating from the FBS in culture medium.

### Cell cytospin and May–Grünwald–Giemsa staining

Cytospins and staining of the cell culture pellets, mentioned above, were prepared by methodology previously described in Oliveira *et al*^17^. Briefly, MAT and SAT SVF cells and PBL were cytocentrifuged at 1000 rpm in a Shandon Cytospin 3 for 5 min. After fixation in methanol, some slides were stored at − 20 °C for future analysis, and others were incubated with May-Grünwald stain for 15 min followed by 5% Giemsa stain for 30 min (both reagents from Merck). After washing with distilled water, slides were dried and mounted with Entellan® (Merck). Cell morphology in cell culture cytospins was analysed to determine cell viability, according to previously described methodology^34^. Consequently, the SAT samples from one animal 12 h after stimuli were excluded from analysis, since cells presented dead cell morphology. In this animal, all cytokines, but IL-8, were undetected in cell cultures, contrastingly to all other samples. IL-8 levels in this sample ranged from 10 (medium) to 100 pg/mL (LPS), which was considerably lower than the medians of the other samples (3402 pg/mL in medium and 6909 pg/mL in LPS culture conditions).

### Immunofluorescence analysis

Immunofluorescence staining was done by a methodology similar to the one described in Teixeira et al.^31^ with modifications. Slides previously stored at − 20 °C were air dried, washed in PBS, and then incubated with 10% FBS (Gibco, MA, USA), 2% BSA (Biowest), 0.5% saponin (Sigma) diluted in PBS in a moist chamber for 30 min at 37 °C. The excess solution was removed, and the sections were then incubated for 1 h at 37 °C with anti-*N. caninum* rabbit antiserum^35^ (kindly provided by Dr. Jose ´ Manuel Costa, Centro de Imunologia e Biologia Parasitária, Porto, Portugal) diluted 1:1500 in 1% BSA and 0.5% saponin in PBS. The slides were then washed with PBS and incubated for 1 h at room temperature with Alexa Fluor® 568 goat anti-rabbit IgG (H + L) secondary antibody (Invitrogen) (1:1000 dilution), Fluorescein isothiocyanate (FITC) anti-bovine CD45 (Clone CC1, Bio-Rad, Kidlington, UK) (1:7,5 dilution), and mouse anti-bovine CD14 (Clone CC-G33, Bio-Rad) conjugated to peridinin-chlorophyll protein-cychrome 5.5 (PerCP-Cy5.5) as described in Oliveira et al.^17^, (1:200 dilution) in 1% BSA and 0.5% saponin in PBS. Slides were mounted in VECTASHIELD Vibrance® Antifade Mounting Medium with DAPI (Vector Laboratories, Newark, USA).

2D Images were acquired with a single point scanning confocal system LEICA Stellaris 8, based on a motorized inverted Leica DMI8 microscope equipped with a HC PL APO 93x/1.3 STED WHITE Corr glycerol immersion objective, a broad range White Light Laser and fully controlled by Leica LASX software ver. 4.6.1.27508 (Leica Microsystems). The images were recorded at 8-bits sequentially by frame scanning unidirectionally with 1024 × 1024 px using the galvanometer scanner at 400 Hz (pixel dwell time of 1.575 μs) resulting in images with a pixel size of 0.122 μm, covering an area size of 125 μm × 125 μm. The DAPI was excited with a 405 nm laser line with a laser power of 2%, and emission was collected on a Leica HyDS1 detector (in analogue mode) with a 420–486 nm collection window (gain of 43.1). Reflected light was acquired in the same sequential scan as DAPI using the 580 nm laser line, in reflection mode, with a power of 2%, and a Leica HyDS3 detector (in analogue mode) placed under the laser line with a 575–585 nm collection window (gain 3.3). CD45, conjugated to FITC, was excited with a 496 nm laser line with a laser power of 8%, and emission was collected on a Leica HyDX2 detector (in digital mode) with a collection window of 506–563 nm (gain of 73), line average of 6, and frame average of 2. Anti-rabbit labelled with Alexa Fluor™ 568 was excited with a 569 nm laser line with a laser power of 3.01%, and emission was collected on a Leica HyDX4 detector (in digital mode) with a collection window of 585–665 nm (gain of 12.9), and a line average of 8. To image the CD14 conjugated with PerCP-Cy5.5, the 496 and 687 nm laser lines were used in combination, both at 8%, and emission was collected on a Leica HyDR5 detector (in digital mode) with a collection window of 697–830 nm (gain of 137.7) with line average of 6, and frame average of 2. 3D images were acquired in the same conditions described for 2D images, with a z-step size of 0.333 μm (z-range of 5.28 μm). For all images, the pinhole size was 151.9 μm, calculated at 1 AU for 580 nm emission.

Representative images were prepared using Fiji/ImageJ 1.54f^36^. Brightness and/or contrast adjustments were made for each channel independently by defining the minimum and maximum displayed intensity values. For all 2D images, the following brightness and/or contrast adjustments were made: DAPI: min of 10, max. of 120; FITC: min.—0, max.—15; Alexa Fluor™ 568: min.: 0, max.: 154; PerCP-Cy5.5: min.: 0, max.: 20; reflected light: min.—3, max.—161. A Median filter of 1 pixel was applied to the DAPI, FITC, and PerCP-Cy5.5channels. For all 3D images, the following brightness and/or contrast adjustments were made: DAPI: min of 10, max. of 62; FITC: min.—0, max.—24/12; Alexa Fluor™ 568: min.: 6, max.: 28; PerCP-Cy5.5: min.: 2, max.: 15.

### Cytokine quantification

Quantification of cytokine levels in cell culture supernatants of SVF cells and PBL was done by sandwich ELISA. Assessment of IL-1β, IL-6 and IL-8 was done using IL-1β Bovine Uncoated ELISA Kit (Invitrogen, Waltham, MA, USA), Bovine IL-6 DuoSet ELISA kit (R&D systems, Minneapolis, MN, USA) and Bovine IL-8 (CXCL8) ELISA kit (MabTech, Nacka Strand, Sweden), respectively, according to the manufacturers’ protocols with minor modification as previously described^37^. As the levels of IL-6 and IL-8 in the SVF cells supernatants exceeded the higher limits of the corresponding ELISA Kits (1000 pg/mL for IL-6 and 800 pg/mL for IL-8), all samples were diluted 1/10 (supernatants of 4 h cultures) or 1/50 (supernatants of 12 h cultures) in PBS with 0.05% Tween-20 and 1% free fatty acid BSA (Biowest) for these cytokines evaluation. Additionally, PBL samples from some experiments were also analysed undiluted for the cytokine IL-6. IL-10 was quantified following a protocol previously described^38^ with major modifications. Briefly, 96-well half-area clear flat bottom polystyrene high bind microplates (Corning) were coated with 1 μg/mL mouse anti-bovine IL-10 mAb (clone CC318; Bio-Rad) diluted in PBS and incubated overnight at 4 °C. Well blocking was done by incubation for 1 h at room temperature with 5% BSA (Biowest) diluted in PBS with 0.05% Tween-20. Cell culture supernatants (25 μL) and standards were then plated and incubated for 2 h at room temperature with shaking. For the standard curve, serial dilutions of recombinant bovine IL-10 (Bio-Rad) were done in dilution buffer (PBS with 0.05% Tween-20 and 0.1% BSA) from 1000 to 3.9 pg/mL. After washing in PBS with 0.05% Tween-20, biotinylated mouse anti-bovine IL-10 (clone CC320; Bio-Rad) was added at 0.5 μg/mL in dilution buffer and incubated 1 h at room temperature. Streptavidin-HRP (MabTech) was then added (diluted 1/1000 in dilution buffer) after washing in PBS with 0.05% Tween-20, and incubated for 1 h at room temperature. Substrate solution (TMB; MabTech) was added after washing and the reaction was stopped with 1 M H_2_SO_4_. The absorbance of each well was measured at 450 nm in a Multiskan™ FC Microplate Photometer (ThermoFisher Scientific, Massachusetts, USA) using SkanIt Software 3.1, with a 620 nm wavelength correction.

Cytokine levels in culture supernatants were highly variable among animals, already noticed in cells incubated with medium only. Therefore, for better visualization of the increases or decreases in cytokine levels, the graphics presented in the main text show fold-change of the cytokines´ levels relative to the levels in the supernatants of cells incubated with medium alone. This was done by dividing the value obtained in each stimulus condition (NcT 10:1, NcT 5:1, NcT 1:1, FK NcT or LPS) by the value obtained in cells incubated with medium alone, for each animal. Nevertheless, the data are presented in pg/mL for the cytokines analysed, for each animal, without any normalization in Supplementary Information S1, as that information is also relevant. Each animal is represented by a different symbol colour that is consistent for the graphics presented in the main text and in Supplementary Information S1 and allows animal discrimination. For IL-1β in PBL culture supernatants at 4 h, only the results in pg/mL are presented as five animals had cytokine levels below detection limit in the medium samples and therefore could not be represented as fold change in main figures.

Besides supernatants of cell cultures, we also analysed the supernatants from the wells containing only complete RPMI that were introduced in each independent experiment as control of cytokines that could be present in the FBS serum used in the cell culture medium. When a signal was detected in these samples (only detected in a few cases and with Abs at 450 nm below 0.04), the values were subtracted from all wells of the respective cytokine and experiment. Cytokines were also evaluated in the supernatants of cultures of NcT alone (without cells) as an additional negative control.

The calculated intra-assay coefficient of variation (CV) for IL-1β was 2.37%, for IL-6 was 8.85%, for IL-8 was 1.84% and for IL-10 was 4.50%. The inter-assay coefficient of variation was 13.39% for IL-1β, 12.74% for IL-6, 14.90% for IL-8 and 12.64% for IL-10.

### RNA extraction and cDNA Synthesis

RNA extraction and conversion to cDNA synthesis were performed as previously described in detail^17,28^, with minor modifications. In brief, total RNA was extracted from the 4 h cultures of MAT, SAT SVF cells and PBL stored in QIAzol Lysis reagent, following the manufacturer’s instructions. All samples of extracted RNA were resuspended in nuclease-free H_2_O in a 10 μL volume and were quantified using the Nanodrop® ND-1000 Spectrophotometer (Thermo Scientific). Conversion of RNA (65.43 ng ± 49.40) to cDNA was done with Maxima® First-Strand cDNA Synthesis kit for RT-qPCR (Fermentas, Thermo Scientific) with a PCR program of 25 °C for 10 min, 50 °C for 30 min, and 85 °C for 5 min, as previously described^17,28^, in a TProfessional Basic Thermocycler (Biometra GmbH, Goettingen, Germany). For all cDNA synthesis reactions, negative controls consisting of RNA samples without reverse transcriptase and tubes with no added RNA were included. Samples were stored at − 20 °C for later usage.

### Quantitative reverse-transcriptase PCR analysis (qRT-PCR)

The methodology used for quantitative reverse-transcriptase PCR analysis was similar to the one previously described in Oliveira et al.^17^. In this work, we used real time PCR method for measuring *IL6*, tumor necrosis factor (*TNF*), interleukin 12B (*IL12B*) and interferon gamma (*IFNG*) mRNA expression levels. As reference genes we used emerin (*EMD*), MARVEL domain containing 1 (*MARVELD1*) and ubiquitously expressed prefoldin like chaperone (*UXT*), since these genes were described to be stable in bovine adipose tissue^39,40^. Sequences of each primer, as well as references where primers were previously described, are depicted in Table 1. *IL12B* and *IFNG* primers were designed with primer-BLAST tool^41^*.* For *IL6, IL12B* and *IFNG* mRNA analysis, the PCR reaction was done in a 10 μL final volume with 0.2 μM of each forward and reverse primer, 1 × Kapa SYBR Fast qPCR master mix (Kapa Biosystems Inc, Wilmington, MA, USA) and 1 μL of the synthesized cDNA (previously diluted 1:10 in nuclease-free H_2_O). The PCR program (95 °C for 5 min; 50 cycles of 95 °C for 10 s and 62 °C for 20 s) was followed by a melting curve analysis. For *IL12B* and *IFNG* gene, we additionally sequenced the obtained PCR products to confirm their specificity. *TNF* mRNA expression levels were determined using primers and a TaqMan® probe (Sequences in Table 1) designed for the *Bos taurus* tumor necrosis factor (*TNF*) gene by TIB MOLBIOL (Berlin, Germany). The PCR reaction was done in a 10 μL final volume with 0.2 μM of each forward and reverse primer, 0.3 μM of the probe, and 1 × Kapa Probe Fast qPCR Master Mix (Kapa Biosystem Inc) and 1 μL of the synthesized cDNA (previously diluted 1:10 in nuclease-free H_2_O). The PCR program consisted of 95 °C for 3 min followed by 60 cycles of 95 °C for 5 s and 60 °C for 20 s. All real-time PCR reactions were done in a Rotor-Gene 6000 (Corbett Life Science, Sydney, Australia). For analysis of real-time PCR data, we used the comparative threshold cycle (C_T_) method^42^ to determine each individual relative gene expression values. The values were calculated using the following formula: 2 – ^(CT gene of interest – CT reference gene)^^42^ where the C_T_ of the reference gene corresponded to the geometric average of all the reference genes analysed, as recommended in Vandesompele et al.^43^.

**Table 1:** Sequence of primers and probe used in gene expression analysis.

Gene symbol	Full name	Primers and probe sequences	Amplicon size (bp)	Original reference or GenBank accession number
*EMD*	Emerin	F:GCCCTCAGCTTCACTCTCAGA R: GAGGCGTTCCCGATCCTT	100	^39^
*MARVELD1*	MARVEL domain containing 1	F: GGCCAGCTGTAAGATCATCACA R:TCTGATCACAGACAGAGCACCAT	100	^39^
*UXT*	Ubiquitously expressed prefoldin like chaperone	F: CAGCTGGCCAAATACCTTCAA R: GTGTCTGGGACCACTGTGTCAA	125	^70^
*IL6*	Interleukin 6	F:CCTGAAGCAAAAGATCGCAGA R: ATGCCCAGGAACTACCACAA	204	^37^
*TNF*	Tumor necrosis factor	F: CTCTGGTTCAAACACTCAGGTC R: GCATTGGCATACGAGTCCC P: 6FAM-TCTTCTCAAgCCTCAAgTAACAAgCCg–BBQ	118	NM_173966.3
*IFNG*	Interferon gamma	F: CAAATTCCGGTGGATGATCTGC R: CAGGCAGGAGGACCATTACG	159	NM_174086.1
*IL12B*	Interleukin 12B	F: CCCGCATTCCTACTTCTCCC R: TCCTGAAGATGGGCTGTACTAA	208	XM_010807562.4

*F* forward primer, *R* reverse primer, *P* probe.

### Statistical analysis

For statistical significance analysis of our data, we used non-parametric tests since our sample size is low (n ≤ 13) and not all data followed a normal distribution^44,45^, as determined by Shapiro–Wilk test and Kolmogorov–Smirnov test normality tests. Statistical significance of data was determined by the non-parametric Friedman test with Dunn’s multiple comparisons test (*P ≤ 0.05; **P ≤ 0.01; ***P ≤ 0.001; ****P ≤ 0.0001). Matched analysis was done for each sample, for each condition tested (medium, live and freeze-killed NcT, LPS). All analysis and graphics were done in GraphPad Prism software, version 9.4.0. Analysis of cytokine production was done on pooled data from 10 to 13 animals, obtained in six independent experiments, as indicated in figure legends. Analysis of the mRNA relative expression was done on pooled data from 8 animals, obtained in four independent experiments. “Pooled data” means that data from each animal, regardless the collection occasion, is represented in the same graphic. In all graphics only individual animals are shown (biological replicates), represented by different symbol colours.

## Results

### In vitro interaction of *N. caninum* with bovine adipose tissue stromal vascular cell fraction cells

**Figure 1:**
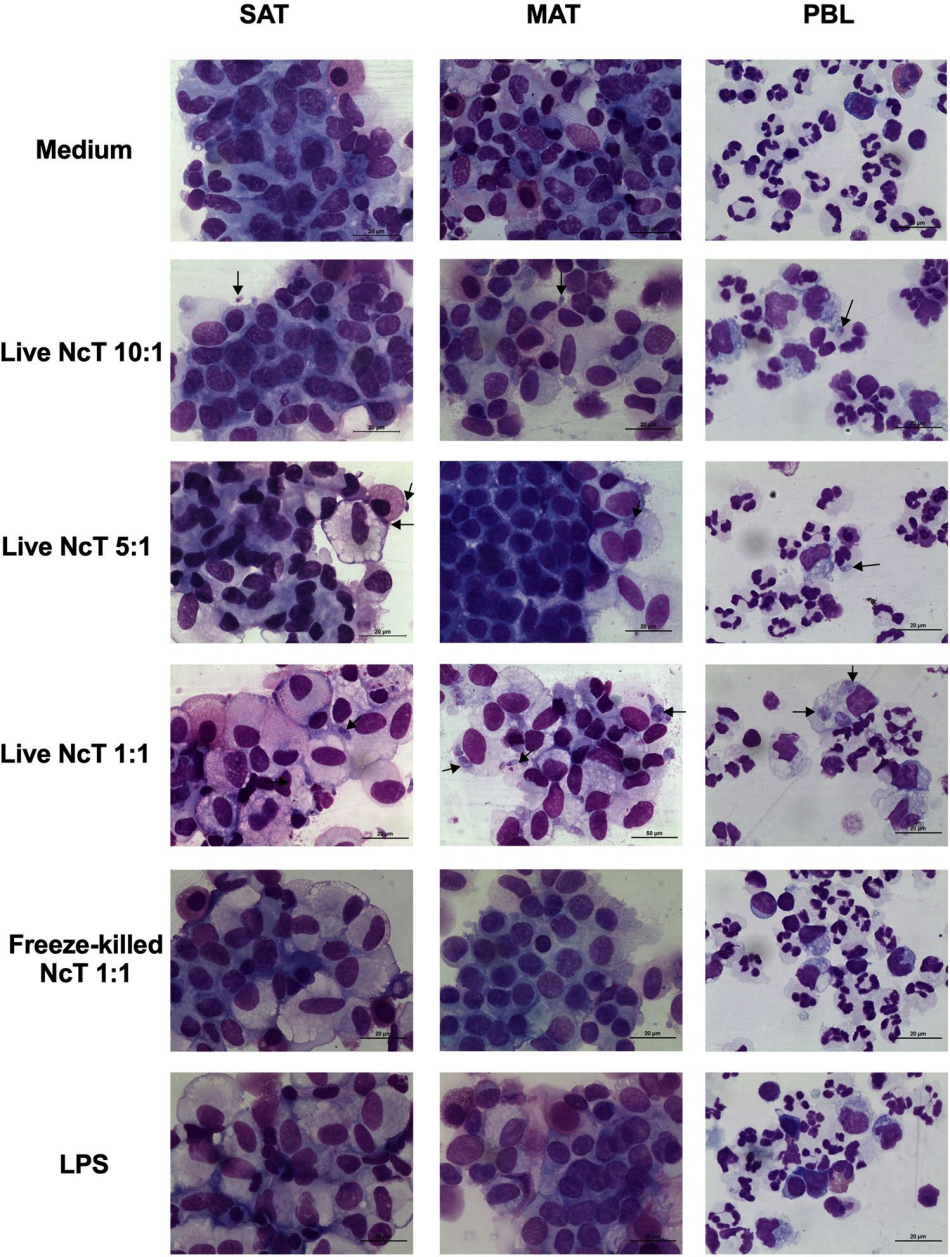
Morphological analysis of cultured bovine adipose tissue stromal vascular fraction cells. Illustrative examples of May-Grünwald-Giemsa staining of stromal vascular fraction (SVF) cells isolated from bovine subcutaneous adipose tissue (SAT), mesenteric adipose tissue (MAT) and peripheral blood leukocytes (PBL) cultured for 12 h alone (medium) or in the presence of live or freeze-killed *N. caninum* tachyzoites (NcT), at a ratio cells/NcT of 10:1, 5:1 and 1:1 or LPS, as indicated. Representative NcT (black arrow) is shown. Scale bar 20 μm. These images are representative examples of micrographs from 4 to 7 animals in MAT, 7 to 12 animals in SAT and 10 to 13 animals in PBL from six independent experiments.

**Figure 2:**
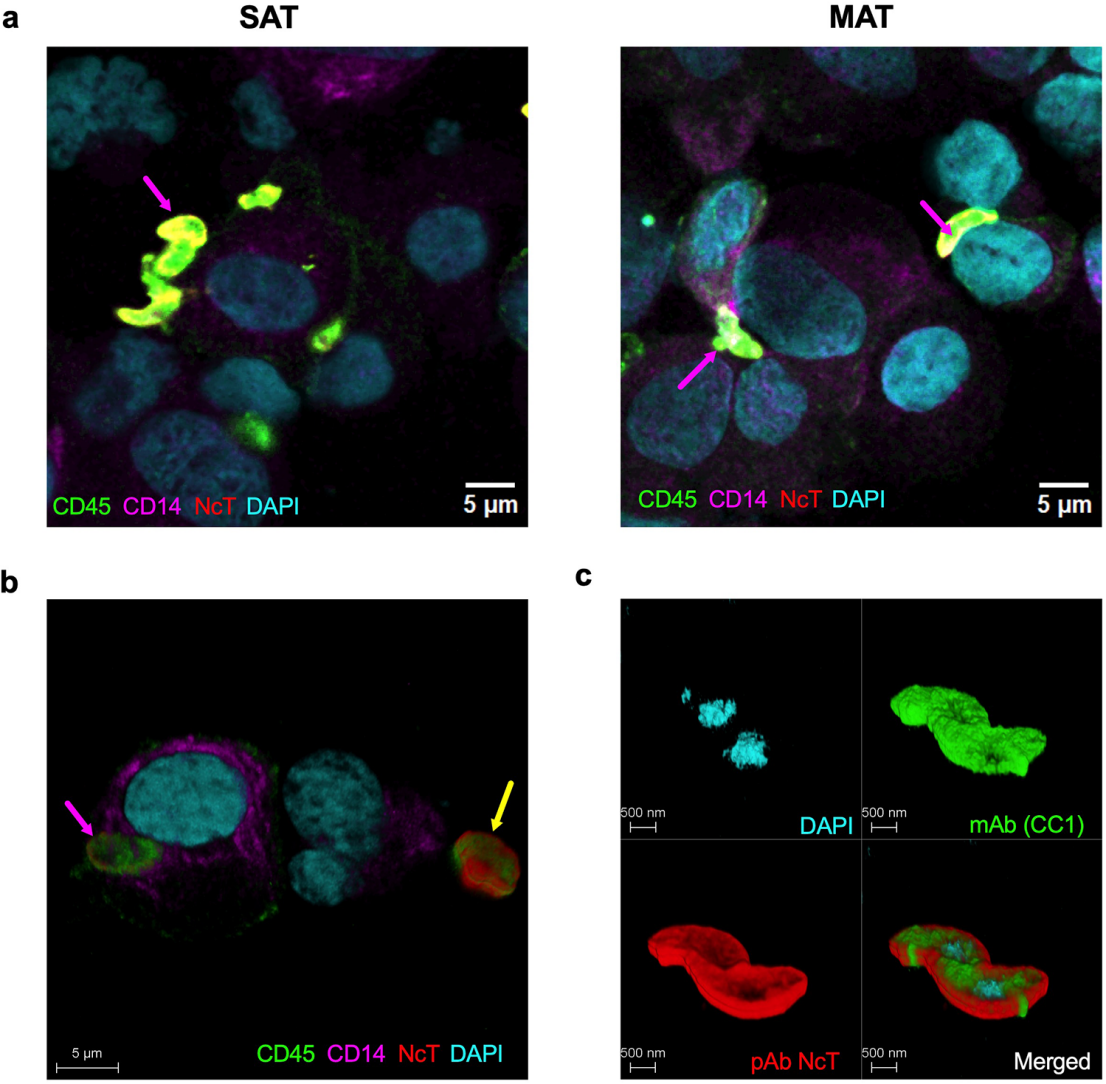
Interaction of *Neospora caninum* tachyzoites with bovine adipose tissue stromal vascular fraction cells. Illustrative examples of confocal immunofluorescence microscopy images of stromal vascular fraction (SVF) cells isolated from bovine subcutaneous adipose tissue (SAT) and mesenteric adipose tissue (MAT), 12 h after in vitro culture with live *N. caninum* tachyzoites. Cell culture cytospins were stained with mAb specific to bovine CD45 conjugated to FITC (green), mAb specific to bovine CD14 conjugated to PerCP-Cy5.5 (magenta) and rabbit pAb specific to *N. caninum* visualized with Alexa Fluor 568 (red). Nuclei are stained with DAPI (cyan). In (**a**), representative images of parasites observed in close interaction with CD45^+^CD14^+^ cells (pink arrows). Scale bar 5 μm. In (**b**) 3D visualization of a parasite located inside a CD45^+^CD14^+^ cell (pink arrow) and a parasite interacting with a CD45^-^ cell (yellow arrow). Scale bar 5 μm. In (**c**) 3D visualization of specific staining of *N. caninum* tachyzoites with rabbit pAb (red) and the binding of the parasite to mAb specific to bovine CD45 (clone CC1) conjugated to FITC (green). Scale bar 500 nm.

SVF cells isolated from MAT and SAT presented different cell types with morphologies alike the ones previously described^17^, such as macrophage-like and mast cell morphology, among others (Fig. 1 and Supplementary Fig. S2). Upon 12 h in culture with the different stimuli (live and freeze-killed *N. caninum* and LPS), both MAT and SAT SVF cells and PBL had morphology compatible with live cells, except one sample of SAT SVF cells that in all conditions (including medium) presented morphology of dead cells and was therefore excluded from the analysis (data not shown). We have previously shown that *N. caninum* could be detected inside murine omental adipose tissue SVF cells displaying macrophage morphology as early as 6 h upon intraperitoneal infection^27^. Similarly, upon in vitro infection of bovine SVF cells, *N. caninum* was found inside or in close interaction with cells displaying macrophage morphology (Supplementary Fig. S2). By immunofluorescence analysis, we observed that cells with such morphology could be CD45^+^CD14^+^ (Fig. 2 and Supplementary Fig. S3), compatible with adipose tissue macrophages^17^, but also CD45^-^ (Fig. 2 and Supplementary Fig. S3). Additionally, we also observed that *N. caninum* parasites were labelled by the antibody specific to bovine CD45 conjugated to FITC (clone CC1), showing a higher fluorescence intensity compared to the CD45^+^ cells (Fig. 2). This is in accordance with preliminary flow cytometry experiments in which the antibody used to identify CD45^+^ cells in SVF cells incubated with *N. caninum* was also binding to the parasite (Supplementary Fig. S4). In May-Grünwald-Giemsa stained cells, we observed the parasite in close interaction with the surface of cells with a round or oval nucleus and with numerous purple granules, a morphology and staining pattern compatible with mast cells^17,46^. More rarely, some images suggested that the parasite could also be located inside these cells (Supplementary Fig. S2).

### IL-6 levels in bovine adipose tissue SVF cell cultures

**Figure 3:**
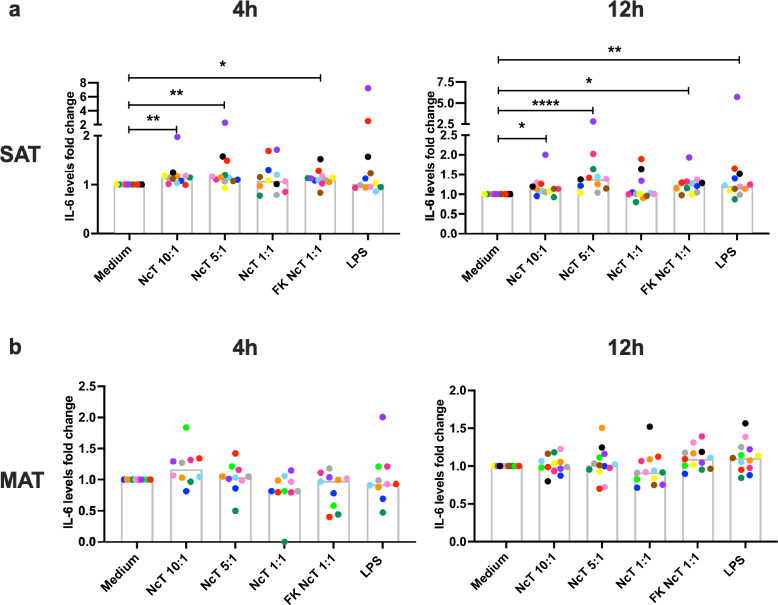
IL-6 production by bovine adipose tissue stromal vascular fraction cells. Fold-change in IL-6 levels in the supernatants of bovine (**a**) subcutaneous adipose tissue (SAT) stromal vascular fraction (SVF) cells and (**b**) mesenteric adipose tissue (MAT) SVF cells cultured for 4 or 12 h in the presence of live or freeze-killed (FK) *N. caninum* tachyzoites (NcT) in cell/NcT ratio of 10:1, 5:1 or 1:1, or LPS, as indicated, relative to levels in the supernatants of cells incubated with medium alone. Each symbol colour represents an individual animal. Bars represent medians of 10–13 bovines per group (at 4 h: n = 12 for SAT, n = 10 for MAT; at 12 h: n = 12 for SAT, n = 13 for MAT), pooled from six independent experiments. Statistically significant differences between different experimental groups are indicated (Friedman test with Dunn’s multiple comparisons test *P ≤ 0.05; **P ≤ 0.01, ***P ≤ 0.001, ****P ≤ 0.0001).

IL-6 is an important cytokine in regulating inflammation in adipose tissue^47^ and previous studies have shown that IL-6 mRNA is upregulated in total bovine adipose tissue upon LPS stimulation^19,20^. Here, both SAT and MAT SVF cells produced large quantities of IL-6 over 12 h of culture (Supplementary Fig. S5) even without stimulation, and therefore dilution of the supernatants was needed for IL-6 quantification, as detailed in the methodology. The same dilution was initially performed in PBL supernatants to allow the direct comparison. However, contrastingly to SVF supernatants, the levels of IL-6 in diluted PBL supernatants were below detection level in the majority of the analysed samples. Therefore, for PBL, we further analysed the undiluted supernatants from the 12 h time point and analysed the *IL6* mRNA expression of cells from the 4 h time point (Supplementary Fig. S6). Comparing the same animals, no significant differences were found between IL-6 levels in the supernatants of MAT and SAT SVF cells cultured for 12 h in medium alone (median values of 14,076 pg/mL in SAT vs 8621 pg/mL in MAT, n = 12; Wilcoxon matched-pairs signed rank test). In undiluted supernatants of non-stimulated 12 h PBL cultures, IL-6 was below detection level in all analysed animals, except one (Supplementary Fig. S6). These results indicate that cells present in the SVF of MAT and SAT produce high basal levels of IL-6. Upon stimulation with both live and freeze-killed NcT, levels of IL-6 were detected increased in the supernatants of cultured SAT SVF cells, at 4 h and 12 h after the parasitic challenge (Fig. 3 and Supplementary Fig. S5). Increased IL-6 levels were also detected in response to LPS 12 h after stimulation (Fig. 3 and Supplementary Fig. S5). Contrastingly, no increased levels of IL-6 were detected in MAT SVF cell cultures in response to the different stimuli (Fig. 3 and Supplementary Fig. S5). These results highlight a distinct responsive capacity of different adipose tissue depots to the used parasitic challenge. Both live and freeze-killed *N. caninum*, and LPS as well, induced increased levels of *IL6* mRNA in cultured PBL 4 h after stimulation (Supplementary Fig. S6). However, no IL-6 increase above detection levels was detected in the supernatants of 12 h PBL cultures upon *N. caninum* stimulation (Supplementary Fig. S6). As increased levels of IL-6 were detected in the PBL cultures stimulated for 12 h with LPS (Supplementary Fig. S6), these results indicate a low responsiveness of circulating leukocytes to *N. caninum* regarding the production of this cytokine.

### IL-8 levels in bovine adipose tissue SVF cell cultures

**Figure 4:**
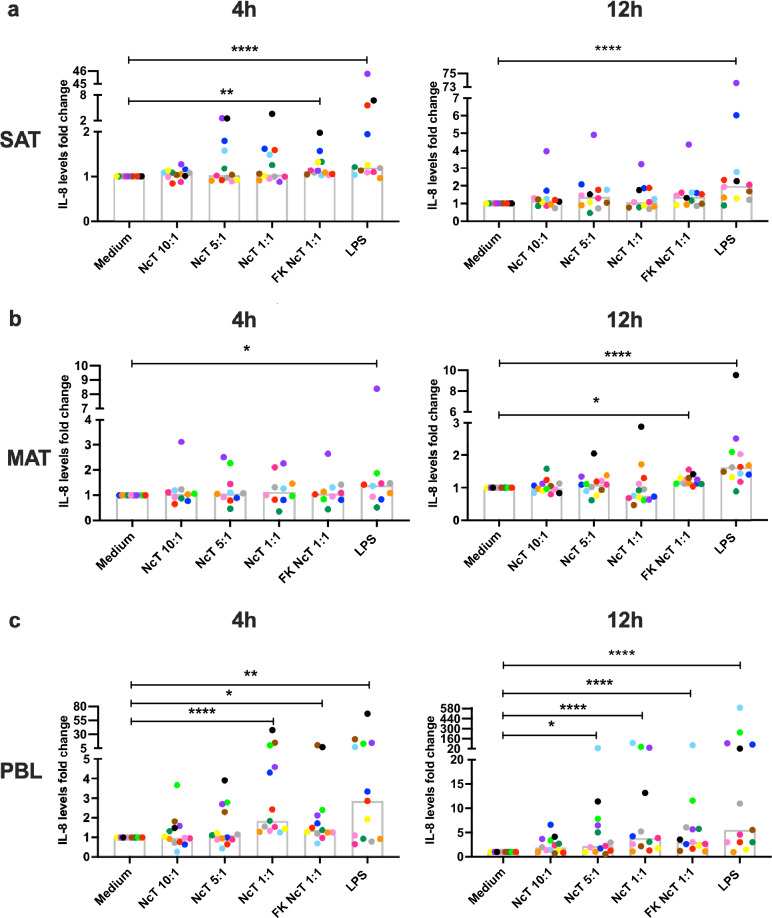
IL-8 production by bovine adipose tissue stromal vascular fraction cells. Fold-change in IL-8 levels in the supernatants of bovine (**a**) subcutaneous adipose tissue (SAT) stromal vascular fraction (SVF) cells, (**b**) mesenteric adipose tissue (MAT) SVF cells and (**c**) peripheral blood leukocytes (PBL) cultured for 4 or 12 h in the presence of live or freeze-killed (FK) *N. caninum* tachyzoites (NcT) in cell/NcT ratio of 10:1, 5:1 or 1:1, or LPS, as indicated, relative to levels in the supernatants of cells incubated with medium alone. Each symbol colour represents an individual animal. Bars represent medians of 10–13 bovines per group (at 4 h: n = 12 for SAT, n = 10 for MAT, and n = 13 for PBL; at 12 h: n = 12 for SAT, n = 13 for MAT and PBL), pooled from six independent experiments. Statistically significant differences between different experimental groups are indicated (Friedman test with Dunn’s multiple comparisons test *P ≤ 0.05; **P ≤ 0.01, ***P ≤ 0.001, ****P ≤ 0.0001).

Another pro-inflammatory cytokine known to be produced in the adipose tissue is IL-8^48^. Similar to IL-6, a high basal production of IL-8 was found for both SAT and MAT SVF cells, as detected in 12 h cultures (Supplementary Fig. S7). The IL-8 levels in non-stimulated SVF cell cultures were higher than the ones detected in non-stimulated PBL cultures. No differences were found between IL-8 levels in the supernatants of SVF cells from the two adipose tissue depots (median values of 3402 pg/mL, 2132 pg/mL and 211.6 pg/mL for SAT, MAT and PBL, respectively; n = 12; SAT vs PBL *P* < 0.0001; MAT vs PBL *P* = 0.0011, MAT vs SAT, *P* = 0.4142, Friedman test with Dunn’s multiple comparisons test). Both MAT and SAT SVF cells responded to LPS with increased production of IL-8, but no significant increase was detected in response to live *N. caninum* (Fig. 4, Supplementary Fig. S7)*.* Nevertheless, a very slight increase in IL-8 levels in the supernatants of SAT and MAT SVF cell cultures was detected in response to freeze-killed *N. caninum* at 4 h and 12 h upon stimulation, respectively (Fig. 4, Supplementary Fig. S7). Contrastingly, increased levels of IL-8 were detected in the supernatants of PBL cultures stimulated with live *N. caninum*, in addition to freeze-killed and LPS stimulation (Fig. 4, Supplementary Fig. S7). These results further highlight the influence of the local environment in the cell responsiveness to *N. caninum*.

### IL-1β levels in bovine adipose tissue SVF cell cultures

**Figure 5:**
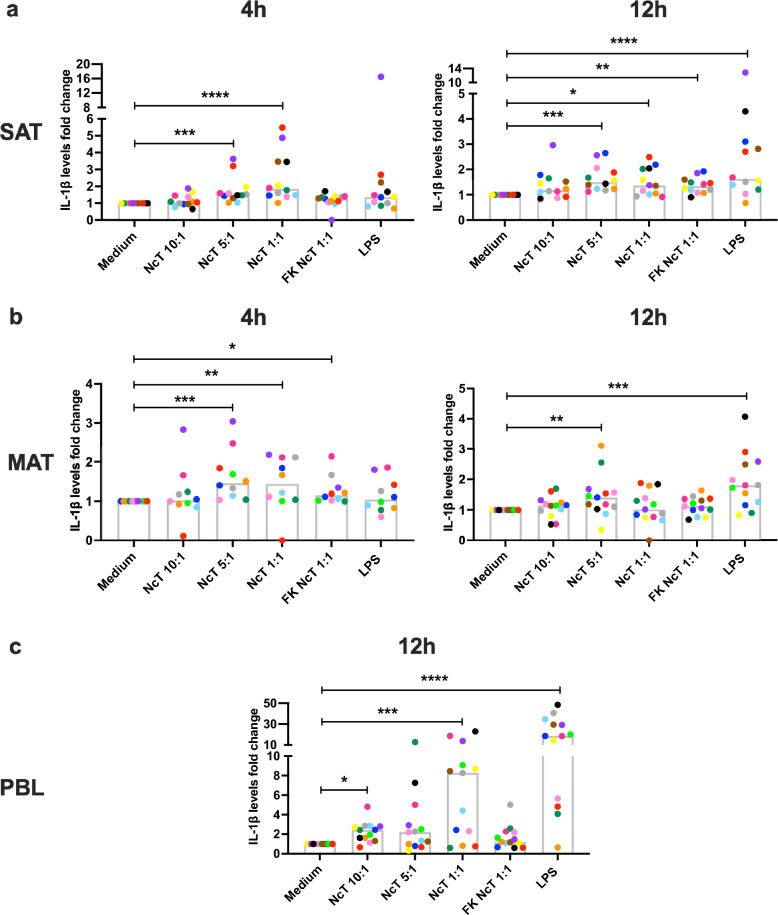
IL-1β production by bovine adipose tissue stromal vascular fraction cells. Fold-change in IL-1β levels in the supernatants of bovine (**a**) subcutaneous adipose tissue (SAT) stromal vascular fraction (SVF) cells, (**b**) mesenteric adipose tissue (MAT) SVF cells cultured for 4 or 12 h and (**c**) peripheral blood leukocytes (PBL) cultured for 12 h in the presence of live or freeze-killed (FK) *N. caninum* tachyzoites (NcT) in cell/NcT ratio of 10:1, 5:1 or 1:1, or LPS, as indicated, relative to levels in the supernatants of cells incubated with medium alone. Each symbol colour represents an individual animal. Bars represent medians of 10–13 bovines per group (at 4 h: n = 12 for SAT and n = 10 for MAT; at 12 h: n = 12 for SAT, n = 13 for MAT and PBL), pooled from six independent experiments. Statistically significant differences between different experimental groups are indicated (Friedman test with Dunn’s multiple comparisons test *P ≤ 0.05; **P ≤ 0.01, ***P ≤ 0.001, ****P ≤ 0.0001).

IL-1β is produced mainly by non-adipocyte cells in the adipose tissue^49,50^**.** The levels of IL-1β were higher in the culture supernatants of non-stimulated SAT compared to PBL when incubated in medium alone (median value of 152.1 pg/mL, 87.93 pg/mL and 13.82 pg/mL for SAT, MAT and PBL, respectively, n = 12; SAT vs PBL *P* = 0.0143; MAT vs PBL *P* = 0.2207; MAT vs SAT, *P* = 0.2207, Friedman test with Dunn’s multiple comparisons test). Live *N. caninum* induced the production of IL-1β by MAT and SAT SVF cells, as well as PBL, as detected 4 h and 12 h after stimulation (Fig. 5 and Supplementary Fig. S8). Freeze-killed parasites also induced the production of IL-1β as detected 4 h and 12 h after stimulation, in the supernatants of MAT and SAT SVF cell cultures, respectively (Fig. 5 and Supplementary Fig. S8). Contrastingly, no significant increase in the IL-1β levels was detected in PBL culture supernatants upon stimulation with freeze-killed parasites (Fig. 5 and Supplementary Fig. S8). LPS induced the production of IL-1β by both SAT and MAT SVF cells 12 h after stimulation (Fig. 5 and Supplementary Fig. S8). In PBL cultures, LPS treatment increased IL-1β levels, detected at both analysed time points (Fig. 5 and Supplementary Fig. S8).

### *TNF* mRNA levels in bovine adipose tissue SVF cell cultures

**Figure 6:**
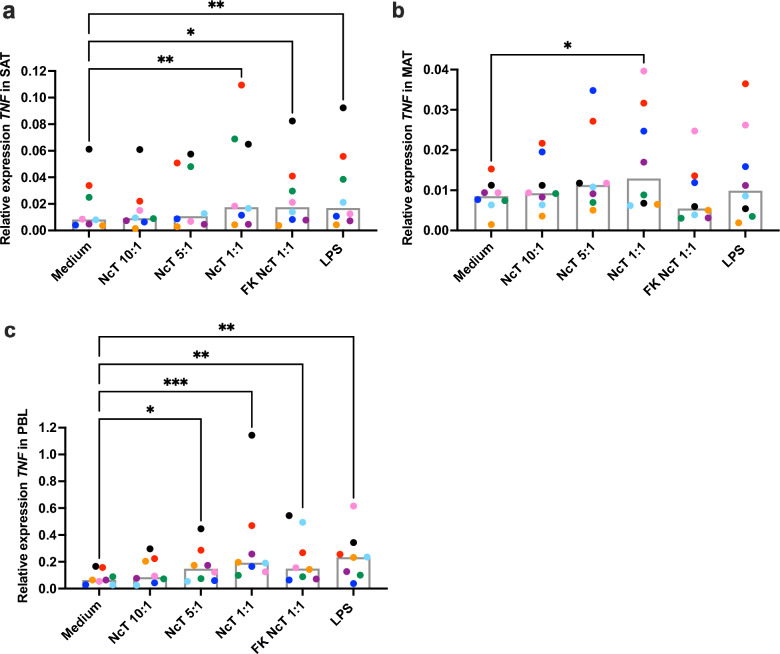
Tumor necrosis factor mRNA expression levels in bovine adipose tissue stromal vascular fraction cells. Relative levels of tumor necrosis factor (*TNF*) mRNA normalized to the geometric averaging of the reference genes emerin (*EMD*), MARVEL domain containing 1 (*MARVELD1*) and ubiquitously expressed prefoldin like chaperone (*UXT*), determined by real-time PCR in bovine (**a**) subcutaneous adipose tissue (SAT) stromal vascular fraction (SVF) cells, (**b**) mesenteric adipose tissue (MAT) SVF cells and (**c**) peripheral blood leukocytes (PBL) cultured for 4 h alone (medium) or in the presence of live or freeze-killed (FK) *N. caninum* tachyzoites (NcT) in cell/NcT ratio of 10:1, 5:1 or 1:1, or LPS, as indicated. Each symbol colour represents an individual animal. Bars represent medians of 8 bovines per group pooled from four independent experiments. Statistically significant differences between different experimental groups are indicated (Friedman test with Dunn’s multiple comparisons test *P ≤ 0.05; **P ≤ 0.01, ***P ≤ 0.001, ****P ≤ 0.0001).

TNF-α is a pro-inflammatory cytokine well known to be expressed in adipose tissue^51^. Here, we decided to evaluate TNF-α expression in cells from the 4 h time point only by PCR due to the low sensitivity of most ELISA Kits available to quantify this cytokine (detection limit ≥ 100 pg/mL)^37^. In cattle, *TNF* mRNA was already detected in the SVF of omental and subcutaneous adipose tissue^15^. Similarly, we detected *TNF* mRNA in non-stimulated MAT and SAT SVF cells at similar levels (Fig. 6), that were approximately 8 times lower than those detected in non-stimulated PBL (median value of 0.008240; 0.008382 and 0.06423 for SAT, MAT and PBL, respectively, n = 8; SAT vs PBL *P* = 0.0179; MAT vs PBL *P* = 0.0035, SAT vs MAT, *P* > 0.9999; Friedman test with Dunn’s multiple comparisons test). In agreement, a previous study showed no differences in *TNF* expression between MAT and SAT whole bovine adipose tissue from Holstein cows^19^. Stimulation with live *N. caninum* induced an increased expression of *TNF* mRNA in MAT and SAT, while freeze-killed parasites only led to detectable increased mRNA levels for this cytokine in SAT (Fig. 6). A significant increase in *TNF* mRNA was also observed in SAT SVF cells in response to LPS (Fig. 6), in agreement with a previous work that showed upregulation of *TNF* mRNA in total bovine adipose tissue upon LPS stimulation^19^. Similar to SAT, increased expression levels of *TNF* mRNA were observed in PBL upon stimulation with live *N. caninum*, freeze-killed *N. caninum* and LPS (Fig. 6). As resistance against neosporosis has been associated with host IL-12 and IFN-γ production^52,53^, we also evaluated mRNA levels of *IL12B* (gene coding for the p40 subunit common to IL-12 and IL-23 cytokines) and *IFNG* in bovine SVF cells and PBL from the 4 h time point in response to medium alone, live *N. caninum* (1:1) and LPS. *IL12B* mRNA levels were below detection level in the majority of SVF samples analysed, and *IFNG* mRNA levels were below detection level in all SVF samples analysed (Supplementary Fig. S9). In PBL, *IL12B* and *IFNG* mRNA were detected in all samples and an increase in *IL12B* mRNA levels was observed upon stimulation with LPS (Supplementary Fig. S9).

### IL-10 levels in bovine adipose tissue SVF cell cultures

**Figure 7:**
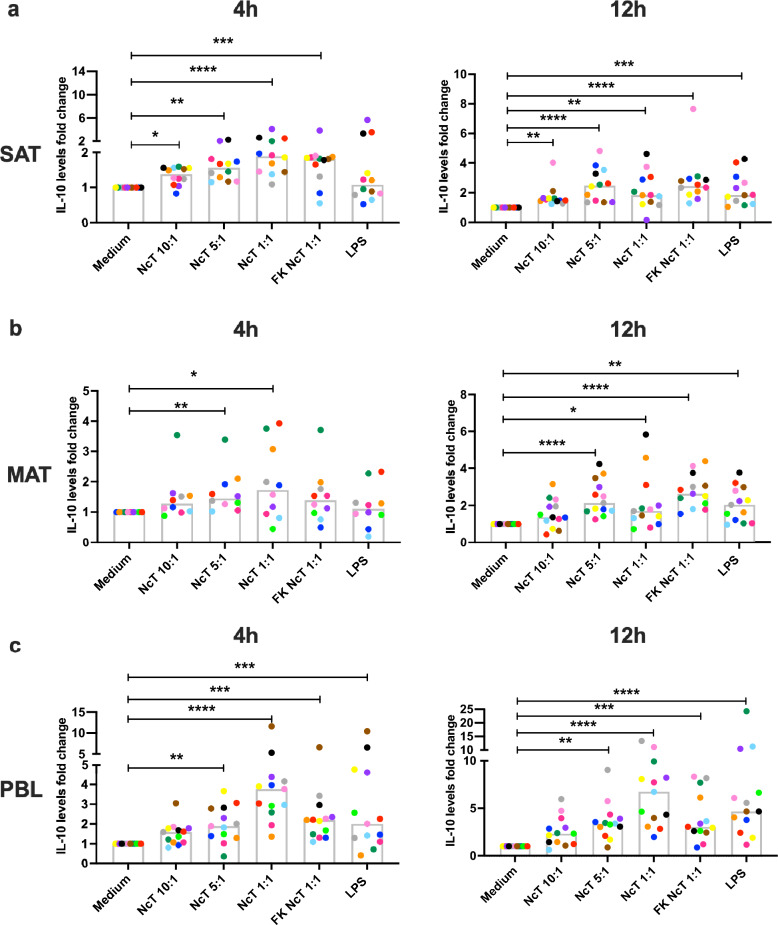
IL-10 production by bovine adipose tissue stromal vascular fraction cells. Fold-change in IL-10 levels in the supernatants of bovine (**a**) subcutaneous adipose tissue (SAT) stromal vascular fraction (SVF) cells, (**b**) mesenteric adipose tissue (MAT) SVF cells and (**c**) peripheral blood leukocytes (PBL) cultured for 4 or 12 h in the presence of live or freeze-killed (FK) *N. caninum* tachyzoites (NcT) in cell/NcT ratio of 10:1, 5:1 or 1:1, or LPS, as indicated, relative to levels in the supernatants of cells incubated with medium alone. Each symbol colour represents an individual animal. Bars represent medians of 10–13 bovines per group (at 4 h: n = 12 for SAT, n = 10 for MAT, and n = 13 for PBL; at 12 h: n = 12 for SAT, n = 13 for MAT and n = 13 for PBL), pooled from six independent experiments. Statistically significant differences between different experimental groups are indicated (Friedman test with Dunn’s multiple comparisons test *P ≤ 0.05; **P ≤ 0.01, ***P ≤ 0.001, ****P ≤ 0.0001).

Having determined that many pro-inflammatory cytokines increased upon *N. caninum* stimulation, we also evaluated the production of the anti-inflammatory cytokine IL-10. The levels of IL-10 detected in basal conditions in SAT and MAT SVF and in PBL culture supernatants were not significantly different (median value of 52.07 pg/mL; 35.46 pg/mL and 49.23 pg/mL for SAT, MAT and PBL, respectively, n = 12; SAT vs PBL *P* = 0.6831; MAT vs PBL *P* = 0.4142; MAT vs SAT, *P* = 0.6831, Friedman test with Dunn’s multiple comparisons test). Upon stimulation with live *N. caninum*, IL-10 levels increased in SAT and MAT SVF cell cultures and in PBL cultures at the two time points analysed (Fig. 7 and Supplementary Fig. S10). Increased levels of IL-10 were also observed in response to freeze-killed *N. caninum* in MAT, SAT and PBL at 12 h and SAT and PBL at 4 h (Fig. 7 and Supplementary Fig. S10) after stimulation. LPS also stimulated IL-10 production by PBL as early as 4 h. This cytokine levels were found significantly increased in response to LPS only at 12 h in SAT and MAT supernatants (Fig. 7 and Supplementary Fig. S10). These results indicate that *N. caninum* consistently stimulates the production of IL-10.

## Discussion

In this work, we show that the SVF of bovine adipose tissue can be a source of pro-inflammatory and anti-inflammatory cytokines in response to both parasitic (*N. caninum*) and bacterial antigens (LPS). Others have previously detected *IL6* mRNA in the stromal vascular fraction of subcutaneous and omental adipose tissue^15^. We show here that both SAT and MAT produce high amounts of IL-6 in vitro even when cultured in medium alone. No differences were found in IL-6 production between these two adipose tissue depots. This is in accordance with a previous report where no differences were found in the *IL6* mRNA levels of MAT and SAT bovine adipose tissue explants from Holstein cows^19^. Whole SAT explants from non-pregnant, non-lactating Holstein cows promptly upregulated *IL6* mRNA expression in response to LPS^19,20^. Here, we show that SAT SVF cells are a source of IL-6 in response to LPS. However, MAT SVF cells did not respond to LPS with increased IL-6 production, in apparent contrast with the reported increased in *IL6* mRNA levels detected in mesenteric adipose explants 2 h upon stimulation with LPS^19^. This might be explained by the fact that we analysed the response of SVF cells and not the whole adipose tissue. Indeed, in the murine model both SVF cells and adipocytes are sources of IL-6^47,51^. Moreover, as we assessed protein and not mRNA levels, which are already high in SVF cells incubated with medium alone, small differences might be harder to detect. We show here that both live and freeze-killed *N. caninum* induced the production of IL-6 by SAT SVF cells. Since increased production of IL-6 in response to *N. caninum* infection by monocyte-derived bovine macrophages was previously reported^54^, adipose tissue macrophages can be one putative source of this cytokine. Contrasting to SAT, IL-6 levels did not increase in MAT SVF cell cultures upon *N. caninum* stimulation. We previously reported that bovine SAT and MAT present different frequencies of immune cell populations^14,17^, that could explain the difference observed in the response as, for example, a higher frequency of macrophages in SAT SVF cells^17^. In PBL cultures, IL-6 was detected in much lower levels than in SAT and MAT SVF cell cultures, likely reflecting the different cell populations present in these tissues. Preadipocytes were shown to be a source of IL-6^55^ in mice and to have high *IL6* expression^7^ in humans. Human adipose tissue derived stem cells have been shown to produce large quantities of IL-6 in vitro^56^, and mesenchymal stem cells derived from bovine adipose tissue were shown to express IL-6^57^. We previously showed that CD45 negative cells constitute 42.9–77.7% and 28.9–77.4% of MAT and SAT SVF cells, respectively^17^. These CD45-negative cells could thus be putative sources of IL-6 in bovine adipose tissue. In mice, IL-6 can promote or suppress adipose tissue inflammation induced by high fat diet, depending on the cellular source and signalling pathway^47^. The biological effects of the IL-6 produced in bovine adipose tissue remain to be determined in future studies.

Another cytokine that we found to be abundantly produced by bovine adipose tissue SVF cells was IL-8. Similarly, SVF cells isolated from human adipose tissue showed production of IL-8 upon incubation in medium alone for 48 h^48^. In accordance with previous work that showed upregulation of *IL8* mRNA levels in bovine SAT explants in response to LPS^20^, we also detected increased production of this cytokine in SAT and MAT SVF cell cultures in response to LPS. No such response was elicited by live *N. caninum* indicating that the response is stimulus-specific. Nevertheless, the high IL-8 levels already encountered in non-stimulated SVF cell cultures could have hindered the detection of putative small increases. Others have previously shown increased *IL8* mRNA levels in bovine umbilical endothelial cells after in vitro infection with *N. caninum*^58^*.* As endothelial cells are present in adipose tissue, they can be a putative source of IL-8 therein in response to freeze-killed parasites. In contrast, a clear increase in IL-8 production was observed in PBL challenged with live and freeze-killed parasites. Human monocyte-derived macrophages produce IL-8 in response to in vitro* N. caninum* infection^59^ and therefore PBL monocytes might be a source of this cytokine.

The pro-inflammatory cytokine IL-1β is also produced by human monocyte-derived macrophages^59^ and bovine macrophages^54^ in response to *N. caninum* infection. We also detected increased levels of this cytokine in PBL and SVF cell cultures challenged with live *N. caninum*. Contrastingly to adipose tissue SVF cell cultures, in PBL no significant increase in IL-1β was detected in response to freeze-killed *N. caninum.* The reasons for this difference remains to be determined, but a possible explanation may rely on distinct cellular sources or distinct mechanisms of IL1-β secretion depending or not on the activation of the inflammasome^60^. In the murine model, others have shown that only live parasites, and not heat attenuated or fixed *N. caninum* parasites or antigens*,* were able to induce IL-1β production by bone marrow-derived macrophages^61^. It would be interesting to assess in the future the cellular sources of IL-1β production in bovine adipose tissue.

In this work, we showed that SVF cells isolated from both MAT and SAT upregulated *TNF* mRNA expression levels in response to live *N. caninum,* similarly to reported observations of increased *TNF* mRNA expression in response to *N. caninum* infection in mouse peritoneal macrophages^62^ and human monocyte-derived macrophages^59^. The pro-inflammatory cytokine TNF-α partially inhibits the growth of *N. caninum* inside primary bovine brain cells *in vitro*^63^ and decreases the number of parasites in rat glial cell cultures^64^. If this cytokine has an effect on the growth of parasites in adipose tissue, it would be interesting to determine in future studies. Similarly to previous studies that showed that soluble *N. caninum* antigens could also induce *TNF* mRNA expression in human monocyte-derived macrophages^59^, we observed increased *TNF* mRNA in SAT SVF and PBL in response to freeze-killed *N. caninu*m, but not in MAT SVF cells. A similar response was observed for LPS. In the murine model, macrophages are one of the main sources of TNF-α in adipose tissue^51^. Since we previously showed that SAT has a higher frequency of macrophages than MAT, the different response of MAT and SAT challenged with freeze-killed *N. caninum* and LPS may be due to differential macrophage proportions. This could also explain the unchanged IL-6 levels in response to the different stimuli in MAT. All cytokines with pro-inflammatory potential evaluated were increased in response to LPS in PBL, as could be expected according to the literature^65^.

IL-12p40 mRNA levels were detected in epididymal white adipose tissue of lean mice^66^, however, in the majority of bovine adipose tissue samples here analysed, they were below detection levels and therefore no conclusions could be drawn regarding the production of this cytokine in response to various stimuli. No conclusion could also be drawn for IFN-γ since gene expression of this cytokine was below detection level in all adipose tissue samples analysed. Later time points might be more suitable for the analysis of this cytokine^28^.

Unlike the production of cytokines with pro-inflammatory potential, the production of the anti-inflammatory cytokine IL-10 was induced, in both adipose tissue SVF cells and PBL, by all the stimuli analysed (live and freeze-killed *N. caninum* and LPS). This production could be important to counterbalance the deleterious effect of produced pro-inflammatory cytokines and avoid host tissue damage^67^. Indeed, others have shown that ex vivo stimulation of bovine adipose tissue explants with TNF-α for 2 h increases *IL10* mRNA levels^68^. We have previously shown increased frequencies of CD4^+^TCRβ^+^-IL-10 producing cells in adipose tissue of mice as early as 24 h after intraperitoneal infection with *N. caninum*^28^. Whether in bovine adipose tissue T cells could also be a source of IL-10 in response to *N. caninum* remains to be determined.

A limitation of our study is that samples were randomly recovered from animals slaughtered for human consumption and not for research purposes, thus we have no access to animals’ clinical history. Also, there is some variation in the age of the animals analysed. The influence that these parameters may have on cytokine production in response to *N. caninum* is therefore unknown. Nevertheless, PBL, MAT and SAT were analysed from the same animals.

In the present study, we sought to improve the characterization of the response to infection induced in bovine adipose tissue SVF cells. It is increasingly recognized that adipose tissue can contribute to the host immune response to pathogens^2^. We show here that bovine MAT and SAT SVF cells respond to *N. caninum* antigens with a cytokine profile different from that observed in cells isolated from blood. Interestingly, the response of MAT SVF cells was less pronounced than the one observed in SAT SVF cells, reinforcing the importance of studying adipose tissue of different anatomical locations to have a more precise knowledge of their contribution to the host immune response to infection. If this difference in response is due to different cell composition or reflects different kinetics of cell infection remains to be determined in future studies.

Overall, our results add to previous studies in the murine model showing that bovine adipose tissue can be a potential contributor to the host immune response to *N. caninum* and it would be an interesting tissue to evaluate in in vivo studies. Our study also contributes to a better understanding of the bovine adipose tissue biology, such as the potential to produce considerable amounts of IL-6 and IL-8. Studies in livestock in the field of adipose tissue biology are increasing, as well as the awareness of the importance of studying this topic to improve animal health^10,69^. It would be interesting in future studies to characterize the cellular sources and functional implications of the detected cytokine production.

### Supplementary Information

Supplementary Information.

## Data Availability

The datasets generated during and/or analysed during the current study are available from the corresponding author on reasonable request.
