# Supplementary material for: Cytokine production by bovine adipose tissue stromal vascular fraction cells upon Neospora caninum stimulation
Source: Sci Rep. 2024 Apr 10;14:8444. doi: 10.1038/s41598-024-58885-z (PMC11006870; doi:10.1038/s41598-024-58885-z)
Supplement: Supplementary file 1 — Supplementary Information. [file 41598_2024_58885_MOESM1_ESM.pdf]

Cytokine production by bovine adipose tissue stromal vascular fraction cells upon *Neospora caninum* stimulation

Bárbara M. Oliveira[1,2,3], Beatriz Sidónio[1,2,3], Alexandra Correia[3,4], Ana Pinto[1,2], Maria M Azevedo[3], Paula Sampaio[3], Paula G. Ferreira[1,2], Manuel Vilanova[3,4], Luzia Teixeira[1,2*]

[1]UMIB-Unidade Multidisciplinar de Investigação Biomédica, ICBAS-Instituto de Ciências Biomédicas de Abel Salazar, Universidade do Porto, Rua de Jorge Viterbo Ferreira, 4050-313 Porto, Portugal.

[2]ITR-Laboratory for Integrative and Translational Research in Population Health, 4050-290 Porto, Portugal.

[3]i3S-Instituto de Investigação e Inovação em Saúde, Universidade do Porto, Rua Alfredo Allen, 4200-135 Porto, Portugal.

[4]ICBAS – Instituto de Ciências Biomédicas Abel Salazar, Universidade do Porto, Rua de Jorge Viterbo Ferreira, 4050-313 Porto, Portugal.

*Address Correspondence to:

Luzia Teixeira, PhD

ICBAS-Instituto de Ciências Biomédicas de Abel Salazar, Universidade do Porto, Rua de Jorge Viterbo Ferreira, 4050-313 Porto, Portugal. Telephone: (+351) 220428109; e-mail: lmteixeira@icbas.up.pt

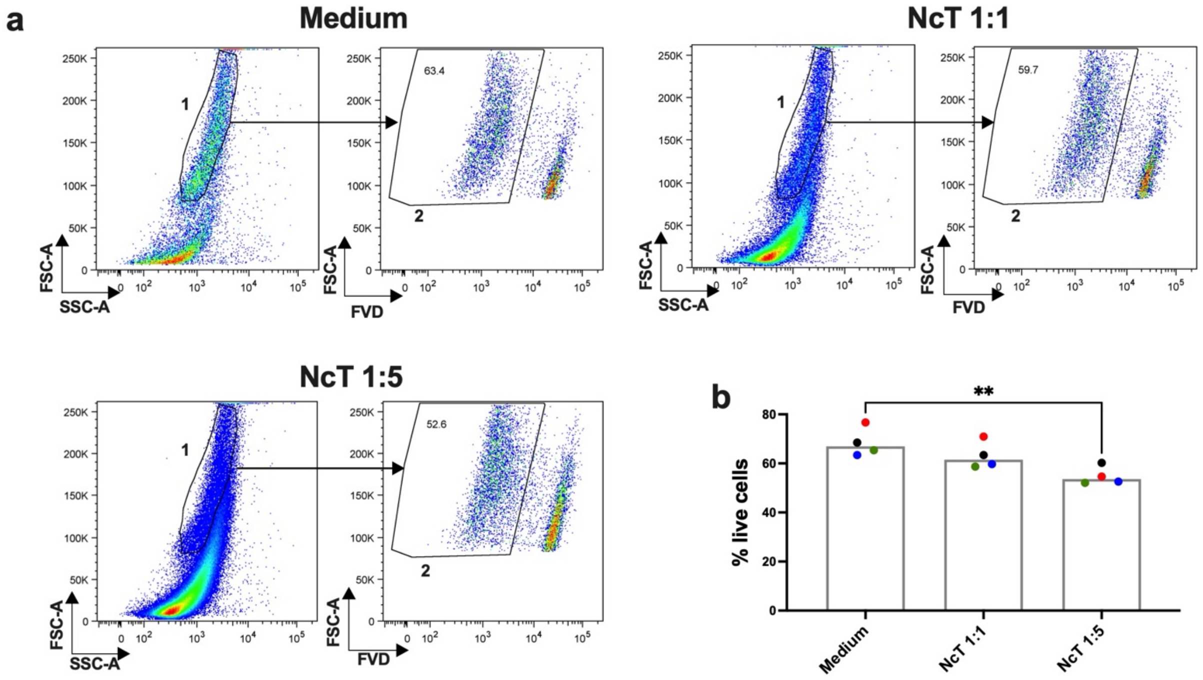

Supplementary Fig. S1. Analysis of live cells in stromal vascular fraction (SVF) of bovine mesenteric adipose tissue (MAT) cultured for 12 h in the presence of live *Neospora caninum* tachyzoites (NcT). **a)** representative example of gating strategy used for selection of live MAT SVF cells after culture in the presence of medium alone or live NcT in cell/NcT ratio of 1:1 and 1:5. Gate number **1** represents selection of cells without debris and avoiding parasites and gate **2** represents selection of live cells; **b)** frequency of live cells in MAT cultured for 12 h alone (medium) or in the presence of live *N. caninum* tachyzoites (NcT) in cell/NcT ratio of 1:1 or 1:5, as indicated. Each symbol colour represents an individual animal. Bars represent medians of 4 bovines per group from one preliminary experiment. (Friedman test with Dunn's multiple comparisons test **P≤0.05).

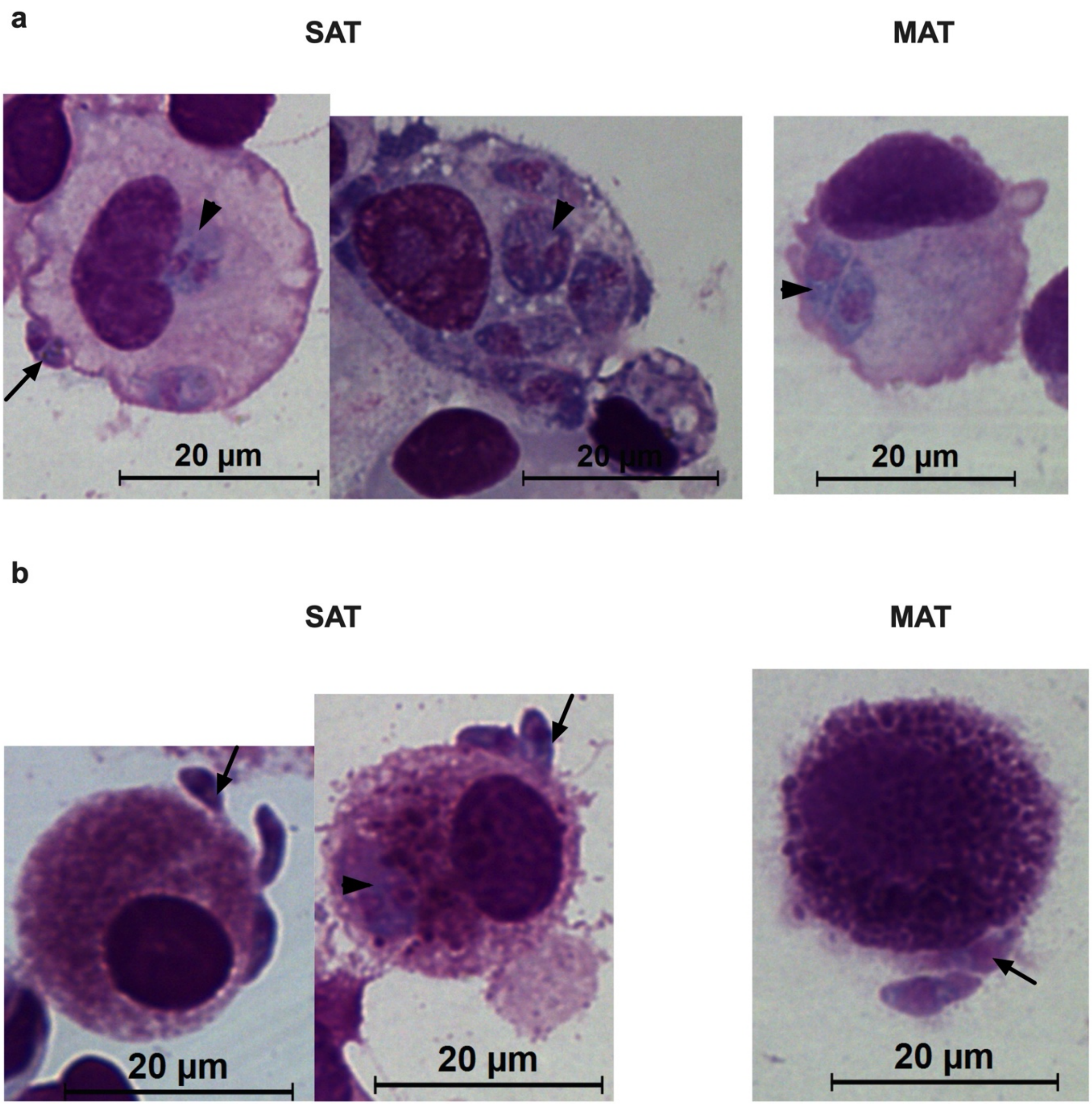

Supplementary Fig. S2. Interaction of *Neospora caninum* tachyzoites with bovine adipose tissue stromal vascular fraction cells. May-Grünwald-Giemsa staining of stromal vascular fraction (SVF) cells isolated from bovine subcutaneous adipose tissue (SAT) and mesenteric adipose tissue (MAT), 12 h after *in vitro* culture with live *N. caninum*. Parasitic forms are observed in close interaction (indicated by arrows) or inside cells (indicated by arrowheads) with **a)** macrophage-like morphology or **b)** mast cell morphology. Scale bar = 20 µm in all micrographs.

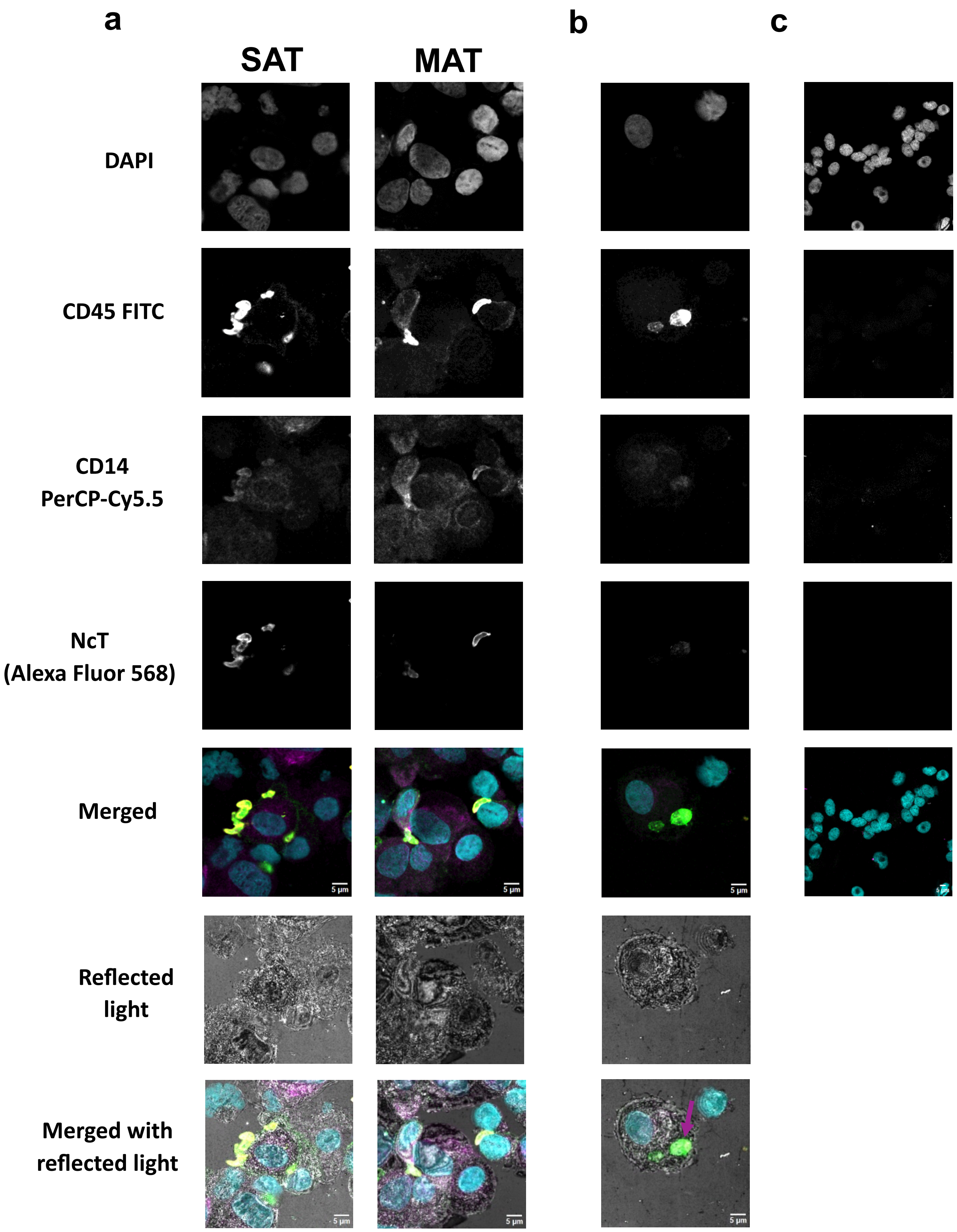

Supplementary Fig. S3. Interaction of *Neospora caninum* tachyzoites with bovine adipose tissue stromal vascular fraction cells. Representative images of confocal immunofluorescence microscopy of stromal vascular fraction (SVF) cells isolated from bovine subcutaneous adipose tissue (SAT) and mesenteric adipose tissue (MAT), 12 h after *in vitro* culture with live *N. caninum* tachyzoites. **a)** Individual channels of images shown in Figure 2a. **b)** Illustrative image of parasites located inside a CD45^{-} cell (pink arrow) and **c)** cells stained only with DAPI. In the merged images, CD45 conjugated to FITC is represented with green, mAb specific to bovine CD14 conjugated to PerCP-Cy5.5 is represented with magenta, rabbit pAb specific to *N. caninum* visualized with Alexa Fluor 568 is represented with red and DAPI, that stains the nuclei, is represented by cyan colour. Scale bar = 5 µm in all images.

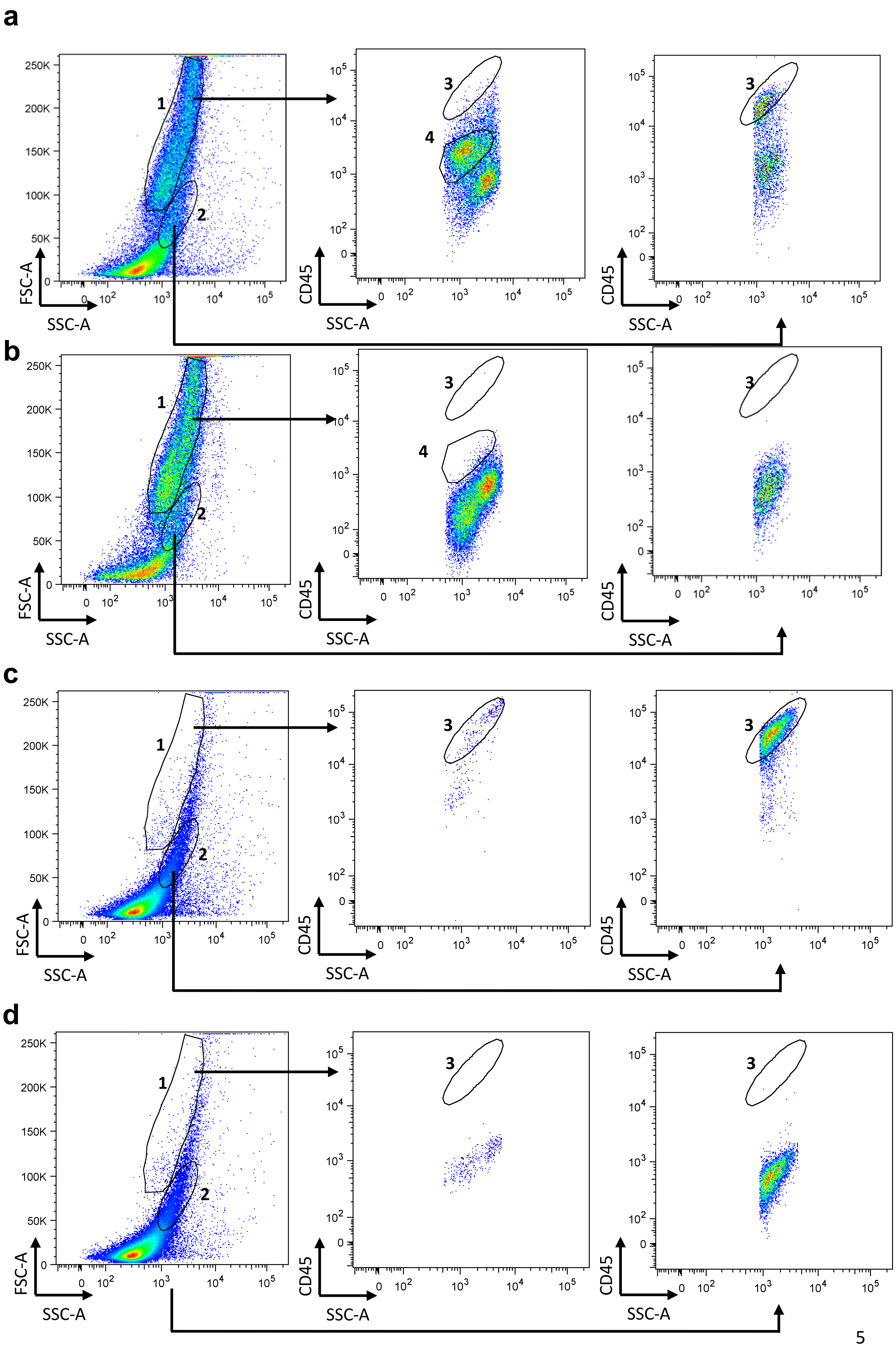
a
b
c
d
FSC-A
SSC-A
CD45
1
2
3
4
250K
200K
150K
100K
50K
0

Supplementary Fig. S4. Representative example of gating strategy used for analysis of CD45 staining in cells isolated from the stromal vascular fraction (SVF) of bovine mesenteric adipose tissue (MAT) cultured in the presence of live *N. caninum* tachyzoites (NcT). SVF cells isolated from bovine MAT were cultured for 12h in the presence of live NcT in 1:1 cell/NcT ratio and either **a)** stained with monoclonal mouse anti-bovine CD45 antibody (clone CC1) conjugated to FITC or **b)** unstained. Parasites (live NcT) cultured for 12h in medium only **c)** stained with mouse anti-bovine CD45 mAb or **d)** unstained. Gate number **1** represents selection of cells without debris and avoiding parasites and gate **2** represents selection of some parasites/cell debris. In gate **3** the location of parasites that stain with the mouse anti-bovine CD45 mAb conjugated to FITC and gate **4** location of cells $CD45^+$. Images are representative of 3 independent experiments.

Flow cytometry methods:

Bovine adipose tissue stromal vascular fraction (SVF) cells and parasites (NcT) were isolated and cultured using the methodology described in the main text. After 12h in culture, cells were stained for flow cytometry analysis by a methodology previously described in Oliveira *el al*[1]. In **Fig. S1**, samples were stained for 30 min with eFluor® 506 Fixable Viability Dye (eBioscience, San Diego, CA) in a 1: 500 dilution in Dulbecco's PBS. After washing, cells were fixed with 2% formaldehyde, washed again and resuspended in Dulbecco's PBS with 2% FBS, 10 mM HEPES and 2 mM EDTA. The frequency of live cells is presented as the frequency of cells within the gate 1 of **Fig. S1**, where we select cells and reduce the inclusion of parasites and cell debris. For **Fig. S2**, MAT SVF cultured cells and NcT were first incubated with purified bovine IgG (Sigma-Aldrich) (100 µg/mL in Dulbecco's PBS with 2% FBS, 10 mM HEPES and 2 mM EDTA) that was used as an Fc blocking reagent for cells. The cells and parasites were then surface stained for 30 min with Fluorescein isothiocyanate (FITC) anti-bovine CD45 (Clone CC1, Bio-Rad, Kidlington, UK). Following incubation, cells were washed, fixed and resuspended in Dulbecco's PBS with 2% FBS, 10 mM HEPES and 2 mM EDTA, as described above. All data acquisition was performed in a FACSCanto™ II system (BD Biosciences, San Jose, CA) with the FACSDIVA™ software (BD) and analysed in FlowJo version 9.9.6. (FlowJo LLC, Ashland, OR).

1. Oliveira, B. M. et al. Characterization of Myeloid Cellular Populations in Mesenteric and Subcutaneous Adipose Tissue of Holstein-Friesian Cows. Sci Rep 10, 1771 (2020). https://doi.org:10.1038/s41598-020-58678-0

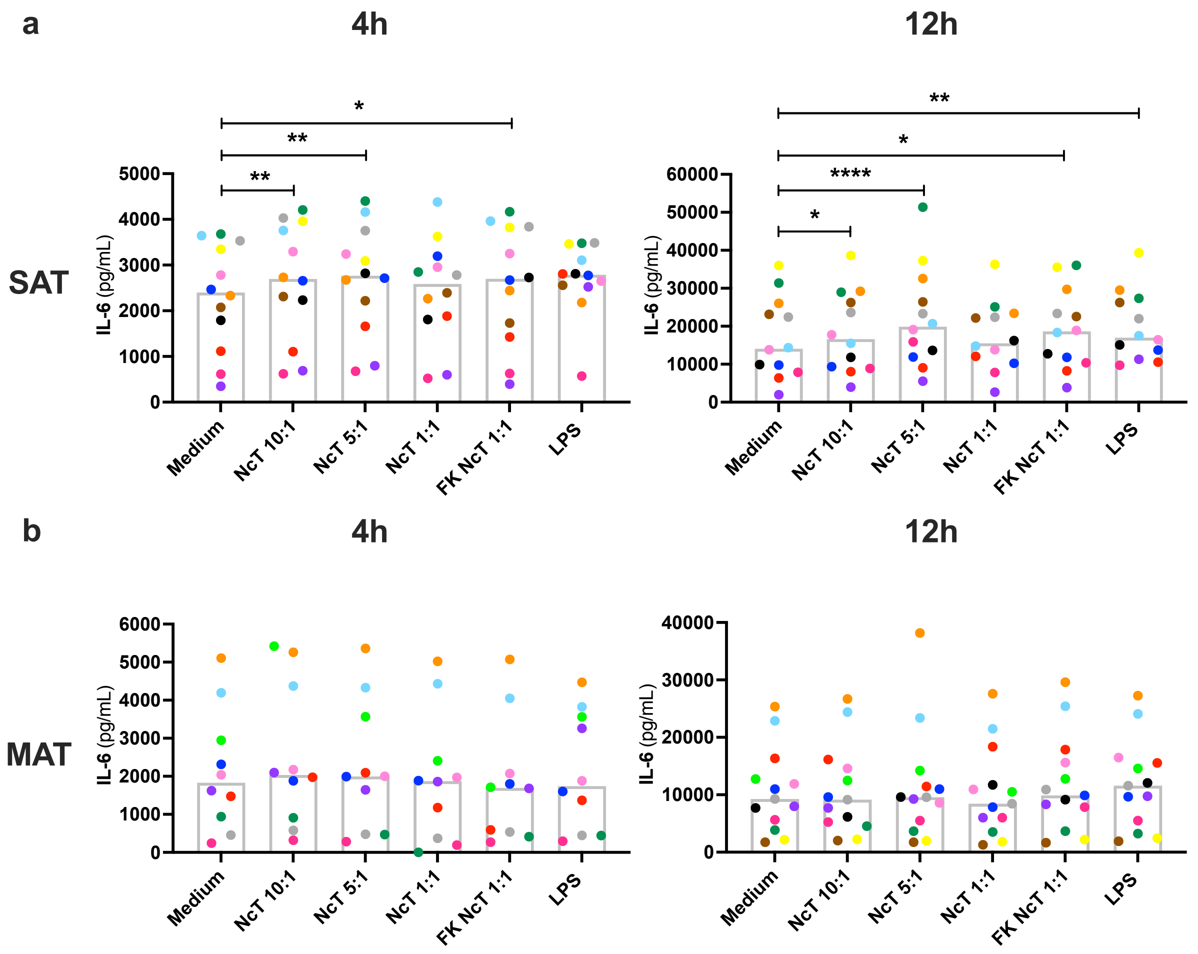

Supplementary Fig. S5. IL-6 levels in the supernatants of bovine adipose tissue stromal vascular fraction cell cultures. IL-6 levels in the supernatants of bovine **a)** subcutaneous adipose tissue (SAT) stromal vascular fraction (SVF) cells and **b)** mesenteric adipose tissue (MAT) SVF cells cultured for 4 or 12 h alone (medium) or in the presence of live or freeze-killed (FK) *N. caninum* tachyzoites (NcT) in cell/NcT ratio of 10:1, 5:1 or 1:1, or LPS, as indicated. Each symbol colour represents an individual animal. Bars represent medians of 10-13 bovines per group (at 4 h: n=12 for SAT, n=10 for MAT and n=13 for PBL; at 12 h: n=12 for SAT, n=13 for MAT and PBL), pooled from six independent experiments. Statistically significant differences between different experimental groups are indicated (Friedman test with Dunn's multiple comparisons test **P*≤0.05; ***P*≤0.01, ****P*≤0.001, ****P≤0.0001).

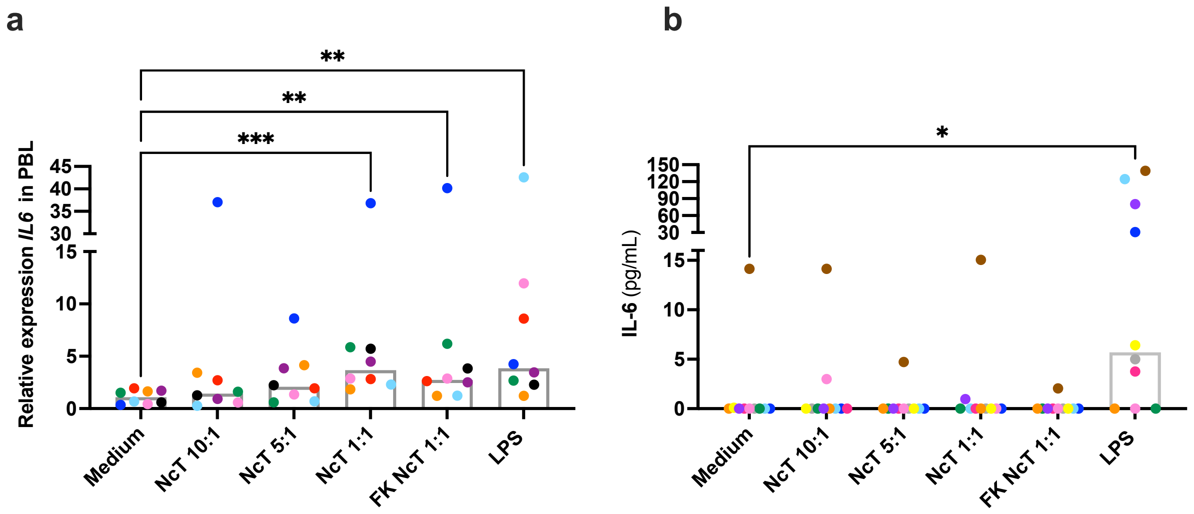

Supplementary Fig. S6. ***IL6*** **mRNA relative expression in PBL cells and IL-6 levels in PBL cultures supernatants**. **a)** Relative levels of interleukin 6 (*IL6*) mRNA normalized to the geometric averaging of the reference genes emerin (*EMD*), MARVEL domain containing 1 (*MARVELD1*) and ubiquitously expressed prefoldin like chaperone (*UXT*), determined by real-time PCR in peripheral blood leukocytes (PBL) cultured for 4 h alone (medium) or in the presence of live or freeze-killed (FK) *N. caninum* tachyzoites (NcT) in cell/NcT ratio of 10:1, 5:1 or 1:1, or LPS, as indicated. Each symbol colour represents an individual animal. Bars represent medians of 8 bovines per group pooled from four independent experiments. **b)** IL-6 levels in undiluted supernatants of bovine peripheral blood leukocytes (PBL) cultured for 12 h alone (medium) or in the presence of live or freeze-killed (FK) *N. caninum* tachyzoites (NcT) in cell/NcT ratio of 10:1, 5:1 or 1:1, or LPS, as indicated. Each symbol colour represents an individual animal. Bars represent medians of 10 bovines per group pooled from five independent experiments. Statistically significant differences between different experimental groups are indicated (Friedman test with Dunn's multiple comparisons test *P≤0.05; **P≤0.01, ***P≤0.001, ****P≤0.0001).

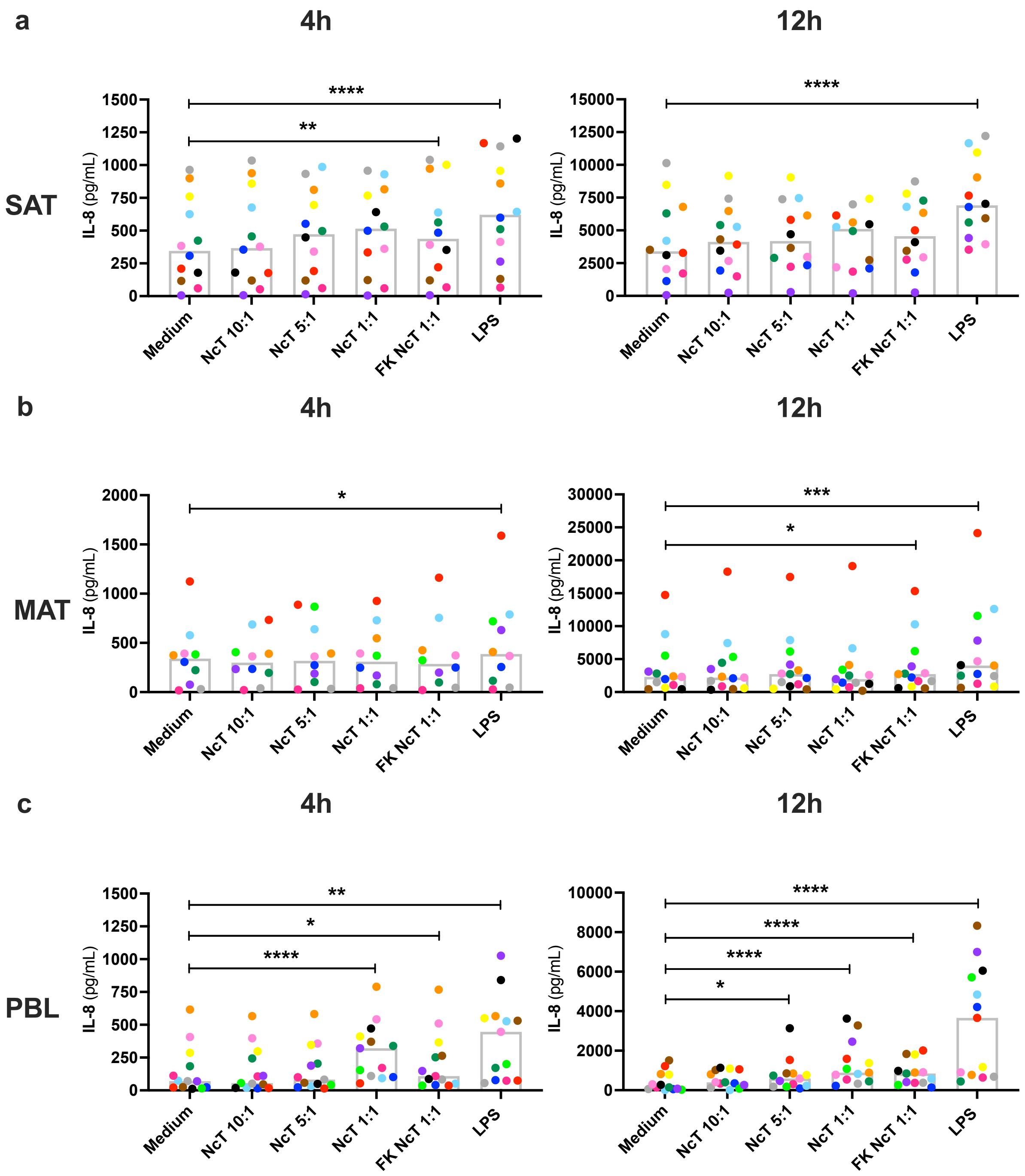

Supplementary Fig. S7. IL-8 levels in the supernatants of bovine adipose tissue stromal vascular fraction cell cultures. IL-8 levels in the supernatants of bovine **a)** subcutaneous adipose tissue (SAT) stromal vascular fraction (SVF) cells, **b)** mesenteric adipose tissue (MAT) SVF cells and **c)** peripheral blood leukocytes (PBL) cultured for 4 or 12 h alone (medium) or in the presence of live or freeze-killed (FK) *N. caninum* tachyzoites (NcT) in cell/NcT ratio of 10:1, 5:1 or 1:1, or LPS, as indicated. Each symbol colour represents an individual animal. Bars represent medians of 10-13 bovines per group (at 4 h: n=12 for SAT, n=10 for MAT and n=13 for PBL; at 12 h: n=12 for SAT, n=13 for MAT and PBL), pooled from six independent experiments. Statistically significant differences between different experimental groups are indicated (Friedman test with Dunn's multiple comparisons test *P≤0.05; **P≤0.01, ***P≤0.001, ****P≤0.0001).

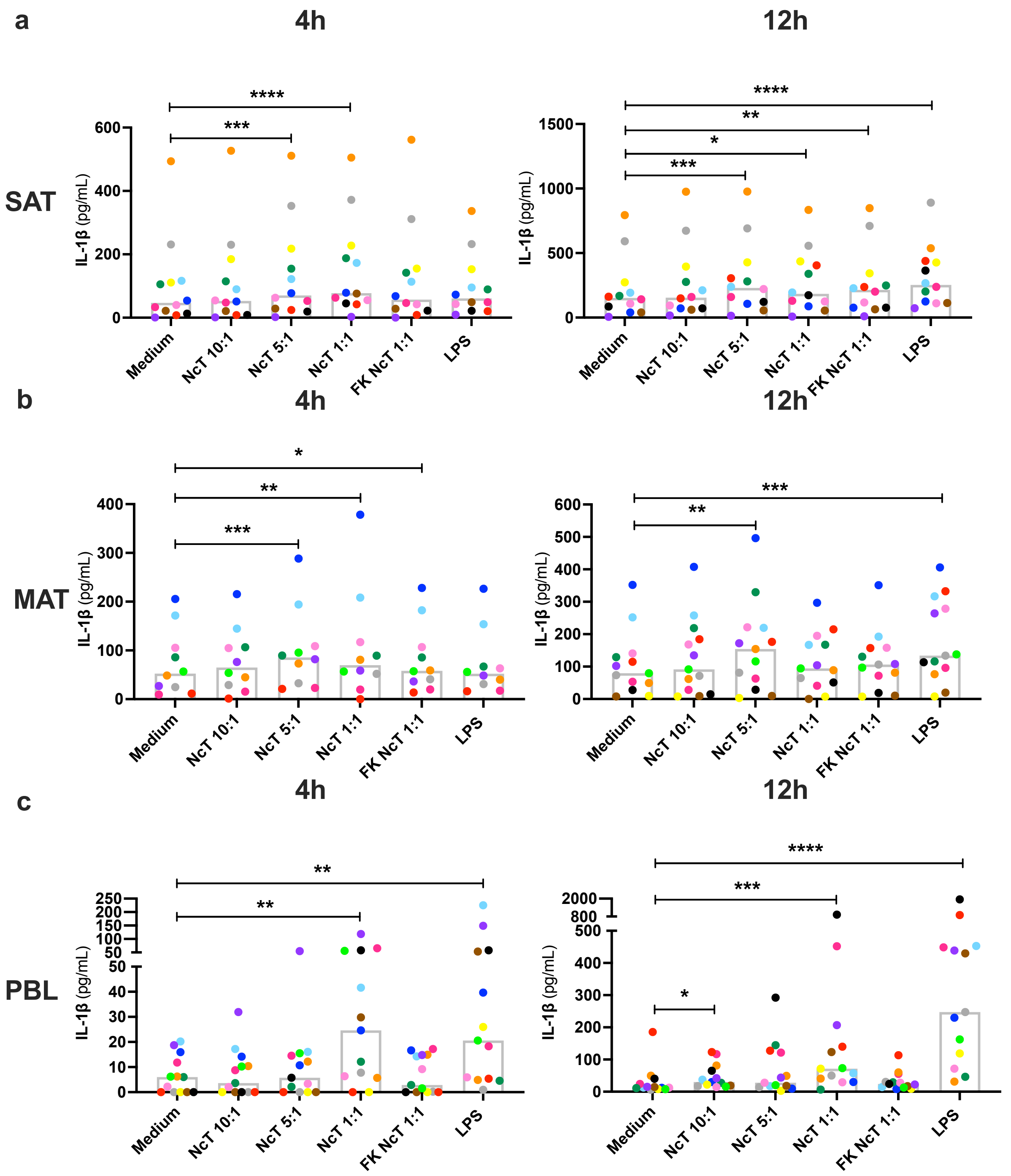

Supplementary Fig. S8. IL-1β levels in the supernatants of bovine adipose tissue stromal vascular fraction cell cultures. IL-1β levels in the supernatants of bovine **a)** subcutaneous adipose tissue (SAT) stromal vascular fraction (SVF) cells, **b)** mesenteric adipose tissue (MAT) SVF cells and **c)** peripheral blood leukocytes (PBL) cultured for 4 or 12 h alone (medium) or in the presence of live or freeze-killed (FK) *N. caninum* tachyzoites (NcT) in cell/NcT ratio of 10:1, 5:1 or 1:1, or LPS, as indicated. Each symbol colour represents an individual animal. Bars represent medians of 11-13 bovines per group (at 4 h: n=12 for SAT, n=10 for MAT and n=13 for PBL; at 12 h: n=12 for SAT, n=13 for MAT and PBL), pooled from six independent experiments. Statistically significant differences between different experimental groups are indicated (Friedman test with Dunn's multiple comparisons test *P≤0.05; **P≤0.01, ***P≤0.001, ****P≤0.0001).

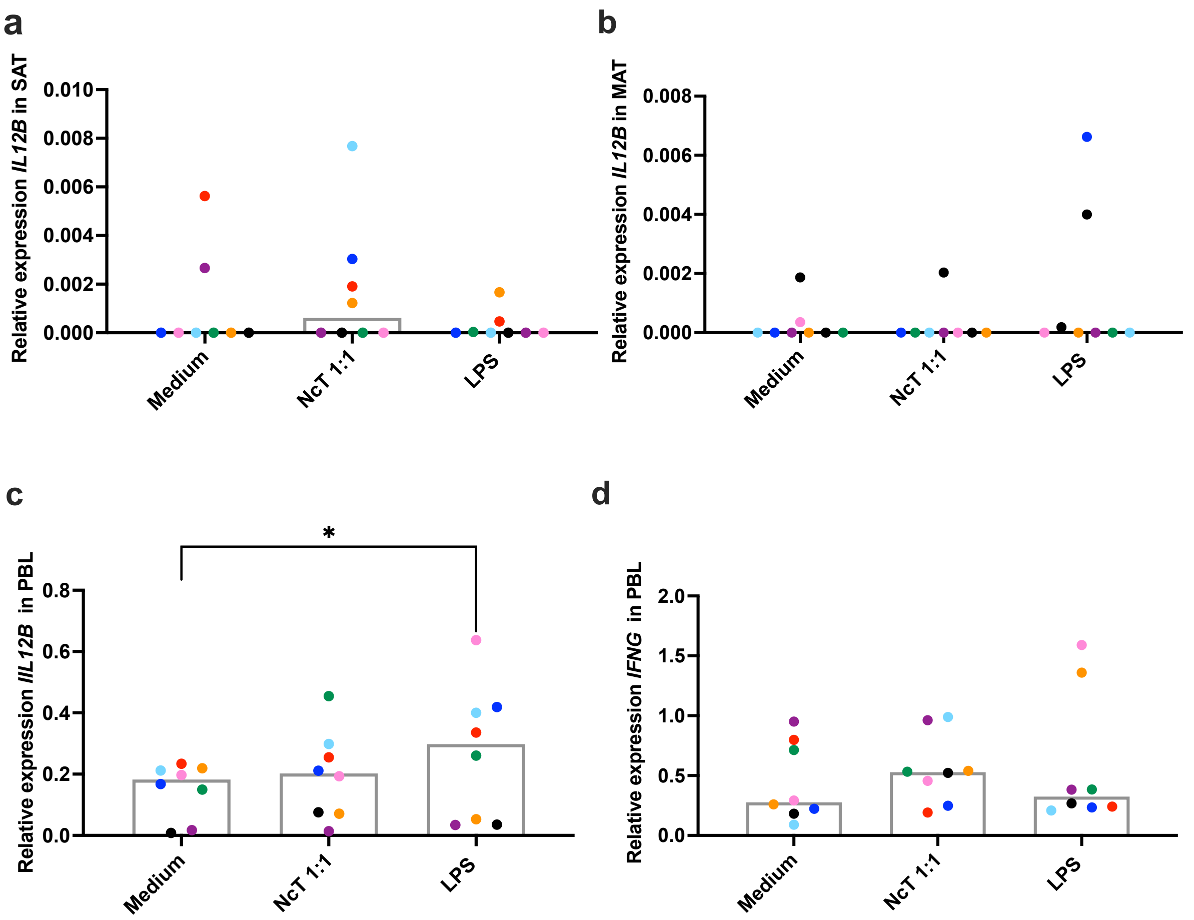

Supplementary Fig. S9. ***IL12*** **mRNA relative expression in bovine subcutaneous and mesenteric adipose tissue stromal vascular fraction cells and** ***IL12*** **and** ***IFNG*** **in peripheral blood leukocytes.** Relative levels of interleukin 12B (*IL12B*) normalized to the geometric averaging of the reference genes emerin (*EMD*), MARVEL domain containing 1 (*MARVELD1*) and ubiquitously expressed prefoldin like chaperone (*UXT*), determined by real-time PCR in bovine **a)** subcutaneous adipose tissue (SAT) stromal vascular fraction (SVF) cells, **b)** mesenteric adipose tissue (MAT) SVF cells and **c)** peripheral blood leukocytes (PBL) cultured for 4 h alone (medium) or in the presence of live *N. caninum* tachyzoites (NcT) in cell/NcT ratio of 1:1, or LPS, as indicated. **d)** Relative levels of interferon gamma (*IFNG*) normalized to the geometric averaging of the reference genes emerin (*EMD*), MARVEL domain containing 1 (*MARVELD1*) and ubiquitously expressed prefoldin like chaperone (*UXT*), determined by real-time PCR in bovine PBL cultured for 4 h alone (medium) or in the presence of live *N. caninum* tachyzoites (NcT) in cell/NcT ratio of 1:1, or LPS, as indicated. Each symbol colour represents an individual animal. Bars represent medians of 8 bovines per group pooled from four independent experiments. Statistically significant differences between different experimental groups are indicated (Friedman test with Dunn's multiple comparisons test **P*≤0.05; ***P*≤0.01, ****P*≤0.001, *****P*≤0.0001).

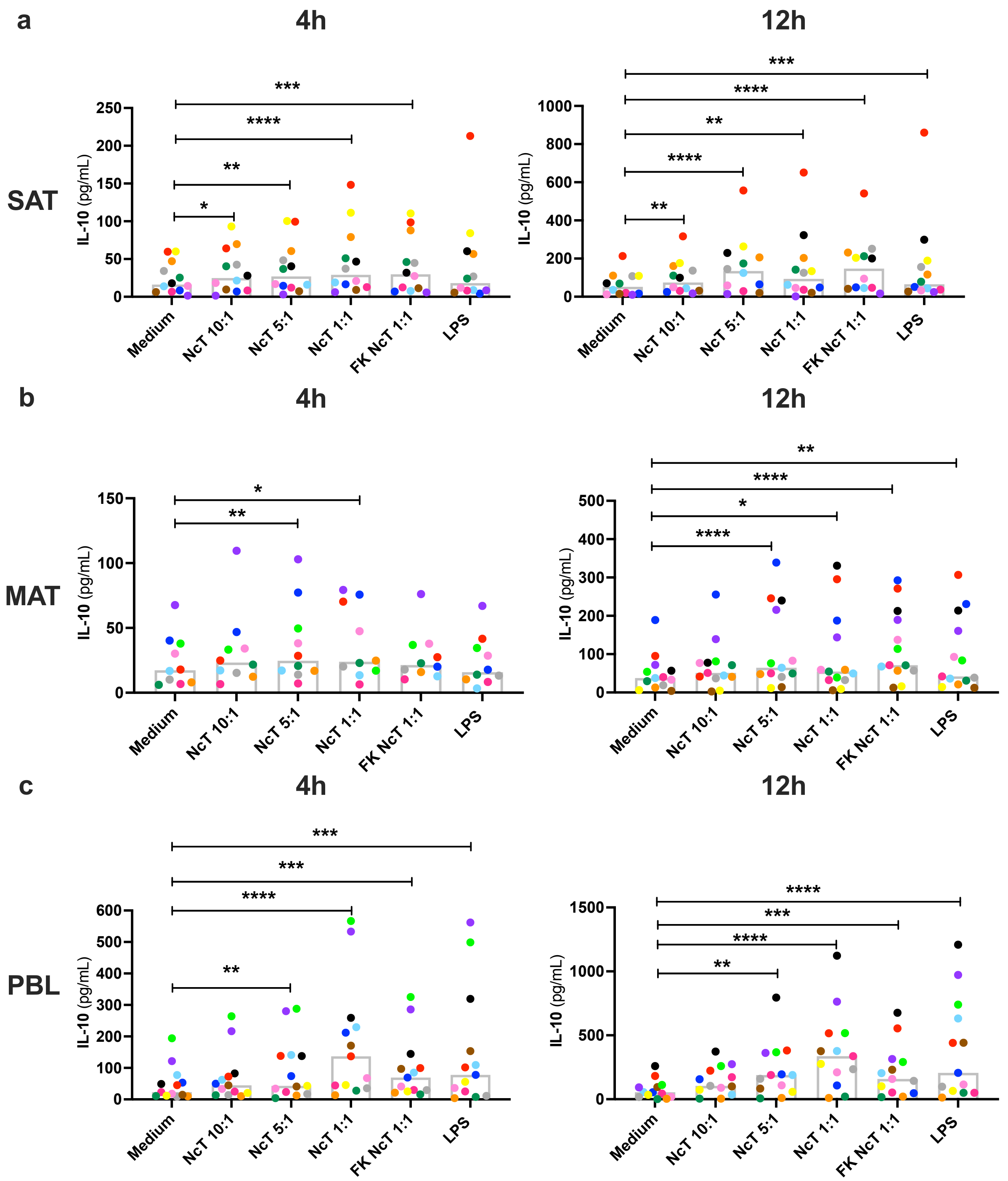

Supplementary Fig. S10. IL-10 levels in the supernatants of bovine adipose tissue stromal vascular fraction cell cultures. IL-10 levels in the supernatants of bovine **a)** subcutaneous adipose tissue (SAT) stromal vascular fraction (SVF) cells, **b)** mesenteric adipose tissue (MAT) SVF cells and **c)** peripheral blood leukocytes (PBL) cultured for 4 or 12 h alone (medium) or in the presence of live or freeze-killed (FK) *N. caninum* tachyzoites (NcT) in cell/NcT ratio of 10:1, 5:1 or 1:1, or LPS, as indicated. Each symbol colour represents an individual animal. Bars represent medians of 11-13 bovines per group (at 4 h: n=12 for SAT, n=10 for MAT and n=13 for PBL; at 12 h: n=12 for SAT, n=13 for MAT and PBL), pooled from six independent experiments. Statistically significant differences between different experimental groups are indicated (Friedman test with Dunn's multiple comparisons test *P≤0.05; **P≤0.01, ***P≤0.001, ****P≤0.0001).

Supplementary Table S1. Age of animals included in the study.

Animal number	Age (months)
**1**	22
**2**	28
**3**	64
**4**	15
**5**	25
**6**	25
**7**	10
**8**	57
**9**	27
**10**	14
**11**	145
**12**	98
**13**	29
